# Example-based learning in heuristic domains: can using relevant content knowledge support the effective allocation of intrinsic, extraneous, and germane cognitive load?

**DOI:** 10.3389/fpsyg.2024.1387095

**Published:** 2024-09-23

**Authors:** Nina Udvardi-Lakos, Marlene Weirich, Julia Asbrand, Alexander Renkl

**Affiliations:** ^1^Educational and Developmental Psychology, Institute of Psychology, Albert-Ludwigs-University of Freiburg, Freiburg, Germany; ^2^Clinical Psychology and Psychotherapy, Albert-Ludwigs-University of Freiburg, Freiburg, Germany; ^3^Clinical Psychology and Psychotherapy, of Children and Adolescents, Humboldt-Universität zu Berlin, Berlin, Germany

**Keywords:** worked examples, example-based learning, exemplifying domain, argumentation, cognitive load theory, skill acquisition

## Abstract

**Introduction:**

Worked examples support initial skill acquisition. They often show skill application on content knowledge from another, “exemplifying” domain (e.g., argumentation skills have to be applied to some contents). Although learners’ focus should remain on the skill, learners need to understand the content knowledge to benefit from worked examples. Previous studies relied on exemplifying domains that are familiar and contain simple topics, to keep learners’ focus on skill acquisition.

**Aim:**

We examined whether using a relevant exemplifying domain would allow learners to acquire both skills and content knowledge simultaneously, or whether relevant content distracts from the main learning goal of skill acquisition.

**Methods and results:**

In a training study with 142 psychology students, we used example-based learning materials with an exemplifying domain that was either relevant or irrelevant for participants’ course outcomes. We assessed cognitive load, declarative knowledge about skills and course-related content knowledge, and argumentation quality. Incorporating relevant content knowledge in worked examples did not reduce learning outcomes compared to a condition using an irrelevant exemplifying domain.

**Discussion:**

Contrary to previous research, the results suggest that worked examples with a relevant exemplifying domain could possibly be an efficient teaching method for fostering skills and content knowledge simultaneously.

## 1 Introduction

Worked examples consist of a problem formulation and solution, typically also including steps leading to the solution (Renkl, 2014, 2021; Sweller et al., 2019). For example, they are often used in mathematics education to show learners how to get from the initial problem to the final solution (Renkl, 2017; Sweller and Cooper, 1985; Tempelaar et al., 2020). Learning from worked examples can reduce learning time and increase performance (e.g., Renkl, 2021; Sweller and Cooper, 1985; van Gog et al., 2019). The worked-example effect thus states that learning from worked examples is more effective than learning by problem-solving during initial skill acquisition (Paas and Van Merriënboer, 1994; Renkl, 2014; Sweller and Cooper, 1985).

Worked examples can also support heuristic skill acquisition, such as argumentation or essay writing (e.g., Hübner et al., 2010; Kyun et al., 2013; Rourke and Sweller, 2009; Schworm and Renkl, 2007; van Gog et al., 2019). Heuristic skills do not have a single correct path to a solution and thus no algorithmic step-by-step approach to teach, but instead require the use of heuristic strategies (Atkinson and Renkl, 2007; Renkl, 2023). Thus, worked examples for heuristic skills need to be structured in a different way than worked examples used for instruction in more well-structured domains, such as mathematics (Renkl et al., 2009). Instead of a clear sequence of steps to follow, heuristic worked examples can show a sequence of heuristic strategies and their steps to approach a problem and find a solution, including tentative and explorative steps (Reiss and Renkl, 2002; Zöttl et al., 2010). Often, such worked examples show the application of a heuristic skill, such as argumentation, using content knowledge (e.g., a controversial topic that is argued about; e.g., Hefter et al., 2014, 2015; Hübner et al., 2010).

Acquiring the content knowledge is usually not part of the learning goals in such studies (Renkl et al., 2009). However, in real classroom learning, students often should acquire both skills and content knowledge. Being able to incorporate relevant content knowledge into worked examples would make instruction more efficient by reducing the time required for learning (Wecker et al., 2016). Furthermore, research suggests that learners can regulate their invested effort in line with their learning goals (de Bruin et al., 2020; Eitel et al., 2020; Sweller et al., 1998). Hence, learners could potentially achieve two learning goals simultaneously by studying worked examples that show skill application using relevant content knowledge. The focus of this study is to investigate whether heuristic worked examples can include to-be-learned content knowledge without hampering skill acquisition.

The worked-example effect is frequently explained by Cognitive Load Theory (CLT; Sweller et al., 1998). According to CLT in its most recent version (Sweller et al., 2019), cognitive load consists of three components: Extraneous load is the load that hinders task performance by introducing unnecessary demands, for example, by sub-optimal presentation of learning materials (Klepsch and Seufert, 2020; Sweller et al., 2019). Intrinsic load describes the load induced by the complexity of the learning task in relation to learners’ prior knowledge (Endres et al., 2022; Sweller et al., 2019). Germane load does not contribute to the overall load, but re-distributes resources to processes relevant for learning (Sweller et al., 2019). Worked examples reduce extraneous load imposed by problem-solving activities that do not promote understanding (e.g., means-ends analysis). Hence, more resources are available for the learning task’s intrinsic load demands. In worked examples, learners can focus on understanding how principles of a skill are applied to example problems and transfer that knowledge to similar problems (Anderson et al., 1997; Renkl, 2014; Scheiter, 2020).

**Figure 1:**
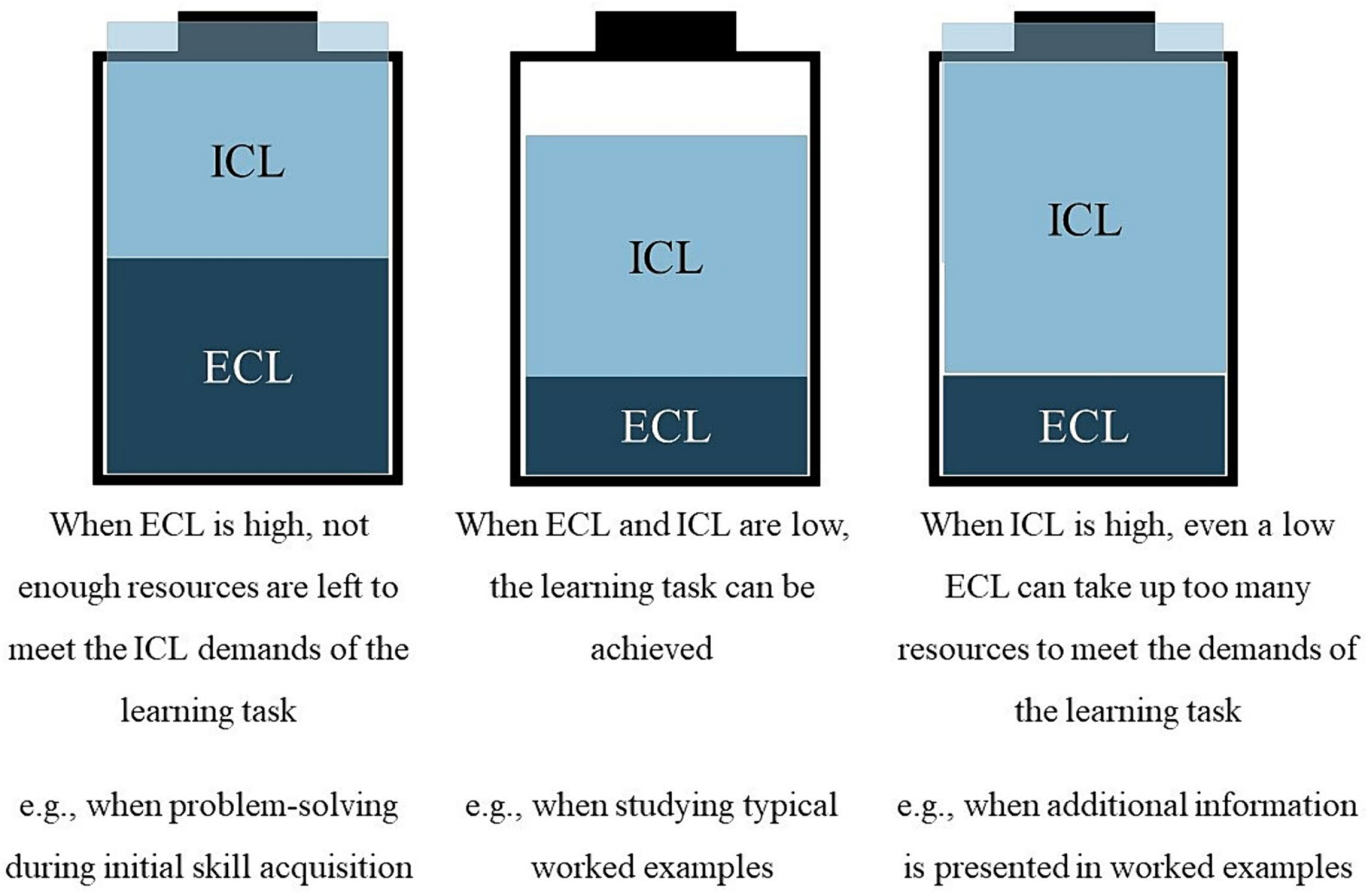
Cognitive capacity is distributed between different load types. ECL = extraneous cognitive load; ICL = intrinsic cognitive load.

While using worked examples reduces extraneous load, instructors should also pay attention to intrinsic load when designing learning materials, as high extraneous load is only overtaxing when intrinsic load is high (Sweller et al., 1998; Sweller and Chandler, 1994; see Figure 1). The main source of intrinsic load is the learning task’s complexity in terms of element interactivity, which refers to the number of elements and the interactions between these elements, which are held in working memory simultaneously (Endres et al., 2022; Sweller, 2011). Worked examples should therefore ideally consist of only the required information needed to understand skill application, without any further to-be-learned information. However, some examples require additional information.

Heuristic worked examples often incorporate two domains, the learning domain (the skill) and the so-called exemplifying domain (content knowledge). For example, when learning how to write an essay (the skill), learners are given a topic to write about, such as a historical event (content knowledge). Worked examples that utilize an exemplifying domain have been termed *double-content examples*, while classical worked examples that only include the learning domain are called *single-content examples* (Renkl et al., 2009). The main focus of learning with double-content examples remains on skill application, while the content knowledge is less relevant (Renkl et al., 2009). Some studies suggest that learning needs to be focused only on one domain to avoid the risk of high cognitive load (Hilbert et al., 2008; Renkl et al., 2009). If learners have to focus on content knowledge as well as the skill, the number of to-be-processed elements in the worked examples increases. The higher element interactivity increases the intrinsic load (Sweller et al., 2019). As intrinsic load and extraneous load are additive, tasks with high intrinsic load could overtax learners even with relatively low extraneous load (Sweller et al., 1998; Sweller and Chandler, 1994). Thus, including relevant content knowledge in worked examples could hinder learning in both domains.

When using double-content worked examples, the learning domain is therefore emphasized over the exemplifying domain. Nevertheless, it is usually important to understand exemplifying contents to some degree, to understand how a skill is applied (Renkl, 2023). For example, when learning from an example essay about a historical topic, the connections between different historical events should be understood. Thus, if exemplifying domain contents are difficult to understand, substantial prior knowledge of the exemplifying domain seems to be necessary to enable learners to benefit from such worked examples (Renkl, 2023; Renkl et al., 2009).

Since worked examples are mostly used to foster skill acquisition, not content knowledge, the exemplifying domain often consists of relatively simple contents and topics familiar but irrelevant to learners. This approach should avoid high load, as learners do not have to invest resources into *learning* the contents (Renkl and Eitel, 2019). Such topics allow focusing on the skill, and do not require substantial prior knowledge to understand and learn from the worked examples; more complex or unfamiliar topics have to be learned as well demand more cognitive resources and may, thus, overwhelm learners and hinder skill acquisition (Renkl et al., 2009; Renkl and Eitel, 2019). Most studies thus far have utilized topics that are simple, familiar, and of little relevance to participants’ goals. For this reason, it is unclear whether content knowledge that is relevant for the learners could be incorporated into worked examples as exemplifying domain without hampering skill acquisition.

Skills training interventions incorporating to-be-learned content knowledge could be more efficient than teaching skills and content knowledge separately (Wecker et al., 2016). Theoretically, double-content examples seem to offer an opportunity to foster skills and content knowledge simultaneously. But how could this efficiency be achieved despite the load elicited by the additional to-be-learned content?

Besides the passive load demanded by a learning task, learners can regulate how much effort they actively invest (Klepsch and Seufert, 2021; Scheiter et al., 2020; Sweller et al., 1998). Additionally, extraneous cognitive load depends not only on the provided instructional design, but also on how it is handled by learners; that is, even with suboptimal instructional designs, cognitive load can be managed by engaging in compensatory processing (Eitel et al., 2020; Mirza et al., 2020).

In their theoretical framework, de Bruin and colleagues described how learners monitor and regulate effort while learning (de Bruin et al., 2020; see also de Bruin and van Merriënboer, 2017). While learners tend to keep their invested effort to a minimum, they can increase effort if they see it as helpful to achieve their goals (Baars et al., 2020; Nückles et al., 2020). With previously used simple, familiar topics, learners may have invested little effort into processing the exemplifying domain and focused mainly on the skill, as they understood the content already (Hefter et al., 2018). If learners perceived the content knowledge of the exemplifying domain to be relevant to their learning goals, they could potentially increase their effort to acquire both the skill and the content knowledge.

A recently developed example-based training aimed at fostering the development of several cognitive skills (e.g., argumentation skills) in psychology students (Udvardi-Lakos et al., 2023). The worked examples were conveyed by videos showing student models, based on the interventions by Hefter et al. (2014, 2015, 2018). In the worked examples, content knowledge from a psychology course was used as exemplifying domain. The participants knew that this content knowledge would be relevant for their course outcomes. This training fostered declarative knowledge about the skills and a higher self-efficacy in using the skills (Udvardi-Lakos et al., 2023). Participants also significantly increased their content knowledge, suggesting that they acquired knowledge about both the skills and the exemplifying domain. However, no control group was used to compare the effects of having simultaneous learning goals (fostering skills and content knowledge) to having these goals consecutively.

In this study, we wanted to replicate these results and address the question of whether relevant content knowledge can be included in worked examples to support the acquisition of heuristic skills and content knowledge simultaneously. Thus, we compared students who receive examples based on course content to students who receive examples with content knowledge irrelevant for passing the course, but with familiar and simple topics. We assume that a relevant exemplifying domain will lead to higher element interactivity than a domain perceived as irrelevant, as the declarative knowledge contents are a part of the learning task and need to be processed in relation to the skill being shown (Endres et al., 2022; Sweller et al., 2019). While the contents may not be more difficult than the irrelevant content, the relevant exemplifying domain should be processed more completely, increasing the intrinsic cognitive load demanded by the worked examples. As heuristic skill acquisition is already demanding, simultaneous learning goals (skill and content knowledge from the exemplifying domain), might cognitively overwhelmed learners (Renkl et al., 2009). Learners would then achieve lower outcomes for their declarative knowledge about the skills, the skills themselves, and the content knowledge being taught.

It might, however, also be possible that learners increase their invested effort to process both the skill and the exemplifying domain if they perceive both as relevant (Klepsch and Seufert, 2021; Taxis et al., 2010). If learners self-manage the cognitive load effectively and actively invest more effort, they may be able to acquire both skills and content knowledge simultaneously. Learners who receive relevant exemplifying domain examples might perceive both a higher intrinsic load and a higher germane load due to investing effort into understanding and elaborating the worked examples to acquire both the skill and the content knowledge. In comparison, learners who receive an irrelevant exemplifying domain may perceive the content knowledge in their examples as less relevant and report a lower cognitive load overall, as they do not invest effort into processing the exemplifying domain (Eitel et al., 2020). For these learners, the content of the exemplifying domain might constitute a form of extraneous load, if they perceive the information as irrelevant and hindering to their goal of acquiring the skill. Thus, it seems prudent to examine the different types of cognitive load associated with the learning materials, to see how learners perceive and regulate the load posed by the learning materials. Our goal is to examine whether the assumptions based on CLT hold true in a sample of students from an undergraduate psychology course.

This study took place in the course “Pedagogy for Psychotherapists” at a German University. The psychology students should acquire both content knowledge and cognitive skills important for psychological practice. The course covers aspects of education and parenting, cultural and social moderating factors, legal and political regulations affecting education, and psychological interventions. The students learn about different viewpoints (e.g., how different factors influence student-teacher-relationships that support social and academic outcomes). Psychology students should incorporate different perspectives or theories in their decision-making. They should learn how to find reliable information, process different viewpoints, and make informed choices. Psychotherapists need these skills when recommending options for their patients. Therefore, we developed a training to foster such skills, based on interventions by Hefter et al. (2014, 2015, 2018).

Our training focused on argumentative thinking skills: These skills are used to weigh various pieces of information and perspectives to come to well-founded conclusions. They involve integrating information and evaluating whether the support for a claim is coherent, relevant, and accurate (Britt et al., 2014). These skills help people to gain a deeper understanding when processing different viewpoints (Felton and Kuhn, 2001; Hefter et al., 2014) and to formulate their own position based on well-supported claims.

Argumentative thinking skills depend on several other components. For example, the understanding that different perspectives should be incorporated into one’s decision-making. This understanding is a part of epistemic beliefs, which refer to an individual’s views on knowledge and the process of knowing (e.g., whether knowledge changes over time; Baxter Magolda, 1996, 2004; Hofer and Pintrich, 1997). Epistemic beliefs form the core of personal epistemologies, influencing beliefs about the self, learning, and instruction, and change through the course of instruction (Baxter Magolda, 1996, 2004). Advanced epistemic beliefs are positively related to learning from different sources and using strategies to judge the validity of different opinions (Kammerer et al., 2015; Mason et al., 2011). Therefore, epistemic beliefs support argumentative thinking skills.

Argumentative thinking is also supported by multiple document literacy (MDL). MDL refers to the ability to combine information presented by different sources and to build a coherent representation of this information in combination with one’s prior knowledge (Bråten et al., 2011; Wineburg, 1991). This representation is necessary to judge different perspectives and come to one’s own conclusion, thus supporting argumentative thinking skills. Therefore, our training incorporated information on argumentative thinking skills, epistemic beliefs, and MDL.

We aimed to replicate findings from a previous study (Udvardi-Lakos et al., 2023), that students show an increase in advanced epistemic beliefs after the training, and we tested whether our training would have a long-term impact on students’ skills using written argumentations. We tested the following hypotheses:

*H*1: After training, participants show more advanced epistemic beliefs.

*H*2: After training, participants score higher on argumentation quality (as an indicator of epistemic beliefs, MDL and argumentative thinking).

Our main focus was to investigate whether students who are exposed to two simultaneous learning goals (having the relevant content knowledge from the “Pedagogy for Psychotherapists” course as exemplifying domain), differ in their learning outcomes compared to students who have the same learning goals consecutively (using topics that are relatively simple and familiar to students as exemplifying domain and providing content knowledge from the course afterwards, so less cognitive load is demanded at any time). We investigated the cognitive load types associated with the learning materials, to see whether simultaneous learning goals led to a higher load compared to consecutive learning goals. We addressed the following exploratory research questions (RQs):

Compared to examples with an irrelevant exemplifying domain, do examples with a relevant exemplifying domain lead to differences in…

RQ1: …intrinsic load,

We expect that examples with a relevant exemplifying domain will lead to higher intrinsic load compared to examples with an irrelevant exemplifying domain. We assume that the relevant exemplifying domain will be perceived as additional information that needs to be processed in relation to the skill being shown, and so leads to a higher element interactivity.

RQ2: …declarative knowledge of skill components and content knowledge,

We expect that examples with a relevant exemplifying domain will not lead to differences in declarative knowledge of skill components and content knowledge compared to examples with an irrelevant exemplifying domain. We assume that learners receiving the relevant exemplifying domain will invest more effort into processing the examples and self-manage the cognitive load effectively, thus showing no detriments from having two simultaneous learning goals.

RQ3: …advanced epistemic beliefs, and.

We expect that examples with a relevant exemplifying domain will not lead to differences in advanced epistemic beliefs compared to examples with an irrelevant exemplifying domain. This expectation is based on the same assumption as RQ2; that learners will self-manage the cognitive load effectively and show no detriments in learning outcomes.

RQ4: …argumentation quality?

We expect that examples with a relevant exemplifying domain will not lead to differences in argumentation quality compared to examples with an irrelevant exemplifying domain. This expectation is based on the same assumption as RQ2; that learners will self-manage the cognitive load effectively and show no detriments in skill acquisition.

## 2 Materials and methods

### 2.1 Sample and design

Participants were undergraduate psychology students taking part in the course “Pedagogy for Psychotherapists” at three German universities. This course is a requirement for entering a psychology Master program leading to psychotherapy training in Germany and covers topics on education and parenting, pedagogical interventions, and social, cultural, political and legal aspects relating to education. Students should understand the issues that children may experience in education and parenting, the context factors involved, and how these influence well-being and psychological health. All students completed the training in an online environment, in the first 2 weeks of the semester as part of their course requirements. They were explicitly told that the training is required to pass their course assessments, which were based on written argumentations about current issues (e.g., issues of inclusion in the German school system); however, the students did not have to consent the use of their data. As consecutive Master programs require high grades in undergraduate courses for entry, a high external motivation could be assumed for students participating in the course.

As we tested both the effects of between-subjects factors (comparing the performance of groups with different exemplifying domains) and learning effects using repeated measures (comparing the performance of individuals across tasks completed at different times), we used a mixed factorial design. A power calculation indicated that a sample of *N* = 52 would be needed to find significant effects in mixed factorial ANOVAs. We assumed a practically relevant, medium effect size of *d* = 0.40 (based on Hattie, 2009) and a power of 0.80.

After being informed of the aim and contents of this research project, 142 students gave informed consent (115 female, 26 male, 1 no response). The training was expected to take around 5 h, including short breaks. All students received the following instructions concerning the contents of the training:

“[…] Within this training, you should acquire important competences for this course. In addition, basic knowledge from developmental and pedagogical psychology will be taught, that will help to prepare you for the first part of the course. […] The training […] is obligatory, as important content knowledge is taught here that you will need for the course. Therefore, please read all information carefully and answer the questions honestly and completely. […]”.

Participants from all locations were randomly allocated to one of two conditions: examples from the relevant exemplifying domain (*n* = 68) or an irrelevant exemplifying domain (*n* = 74). Content knowledge from the “Pedagogy for Psychotherapists” course was used as relevant exemplifying domain as this knowledge was relevant to the participants’ course outcomes (the relation between violence-containing media and aggression in children, the German school tracking system, and the use of grades in schools). These topics were tied into the course materials and matched the learning goals of the course (e.g., “The learners can suggest concrete recommendations to support and improve learning processes in schools and argue for their implementation.”). Participants were explicitly told that the content knowledge from the training would be expected during the course and for the final course exam. Relevance was thus defined as relevant to the course learning goals and course performance. Three topics that were familiar to students and that are relatively simple (the wages of professional athletes, the privatization of the German railway system, and the use of homeopathy) were used as irrelevant exemplifying domain. The topics were taken from three different domains, based on the assumption that students would not be interested in all three topics and thus would perceive these topics as irrelevant, next to their lack of connection to the course content.

### 2.2 Training

**Figure 2:**
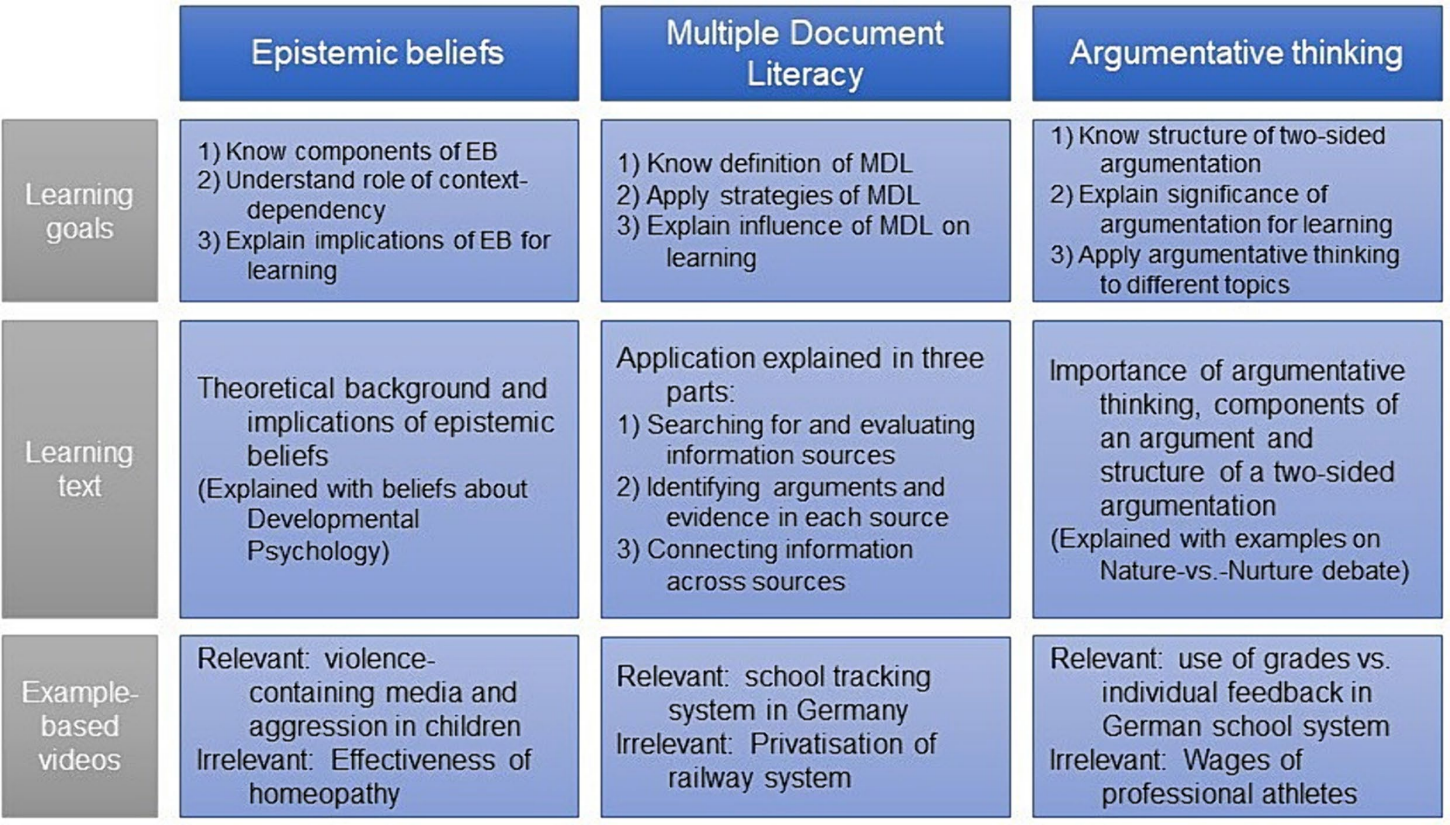
Overview of training components. EB = epistemic beliefs; MDL = multiple document literacy.

The training consisted of three modules: epistemic beliefs, MDL, and argumentative thinking skills. Each module consisted of three parts (see Figure 2 for overview): First, participants were informed about the learning goals. Secondly, participants received an introductory text about the component being trained. In this text, course-relevant content knowledge was used to provide a first example of the given component (e.g., explaining argument structure using an argument about the nature–nurture debate). Learning goals and introductory texts were the same across conditions. Then, participants received a video showing an example case of how this component can be applied, with different exemplifying domains used in the different conditions. The videos were segmented into meaningful sections, each followed by self-explanation prompts (see Supplementary material A for excerpts from the training). This procedure has been found to be effective (Hefter et al., 2015; Udvardi-Lakos et al., 2023).

### 2.3 Instruments and materials

#### 2.3.1 Demographic questions

Participants were asked to indicate their gender, age, and Abitur grade (German university entrance qualification).

#### 2.3.2 Epistemic beliefs

We assessed epistemic beliefs before and after the training with the short version of the German Connotative Aspects of Epistemological Beliefs (CAEB; Stahl and Bromme, 2007). The CAEB consists of 17 pairs of oppositional adjectives, where participants rated how knowledge could be described by the poles of the respective adjective pair (e.g., “objective” vs. “subjective,” “permanent” vs. “unstable,” “vague” vs. “exact”) on a seven-point scale. For this study, participants were asked to rate how well the adjectives fit the sentence ‘Scientific knowledge is …’. This questionnaire was used so that participants could not choose answers based on what they had just learned in the training, but had to apply their understanding of scientific knowledge to rate this concept in relation to the given adjectives. The CAEB has a two-factor structure: Texture and Variability (Stahl and Bromme, 2007). Texture includes beliefs about the structure and accuracy of knowledge. Variability refers to beliefs about the stability and dynamics of knowledge (Stahl and Bromme, 2007). The reliabilities for the two factors were satisfying (see Supplementary material B for reliability scores).

#### 2.3.3 Prior knowledge

Prior knowledge about the three components (epistemic beliefs, MDL, argumentative thinking skills) and the course-related content of the relevant exemplifying domain was assessed before the training with three questions for each construct (see Supplementary material C for items). The first question required general recognition. The second question required participants to name specific concepts. The third question required participants to explain a given concept using 1–2 sentences. The answers were coded on a scale from 0–3 points. Participants could receive a total of nine points for each of the skill components and for the course-related content knowledge tests.

#### 2.3.4 Cognitive load

Cognitive load induced by the learning materials was measured after each module using the questionnaire by Klepsch and Seufert (2020). This instrument assesses extraneous, intrinsic, and germane cognitive load using seven statements that are rated on a seven-point scale (e.g., “This task was very complex,” “During this task, it was exhausting to find the important information”). This scale has been shown to have good validity (Klepsch and Seufert, 2021); we found satisfying reliabilities (see Supplementary material B).

#### 2.3.5 Training duration

We measured training duration as the difference between the start time and submission time in seconds. Participants were given a timeframe of 2 weeks to complete the training, with the option to save and continue anytime during this time period. This approach means that the duration measure gives only a very rough indication of the time actively spent training, as we could not control for breaks (for data protection reasons).

#### 2.3.6 Learning outcomes

##### 2.3.6.1 Declarative knowledge

Participants completed four tests assessing declarative knowledge with items requiring “true” or “false” judgments (skill components each with 15 items, and course-related, relevant exemplifying domain, 30 items) after the training (for example items, see Supplementary material D). The tests all had high reliabilities (all Cronbach’s α ≥ 0.957, see Supplementary material B).

##### 2.3.6.2 Argumentations

Participants were asked to write four argumentations. Writing a good argumentation requires all three components taught in the training: epistemic beliefs (to understand the importance of considering different perspectives), MDL (to integrate various sources), and argumentative thinking skills (to weigh evidence and form a conclusion). The first two argumentations were written before and just after the training session. Participants were given three texts for each argumentation (about 360 words each), explaining different positions and arguments supporting these positions. The third and fourth argumentations took place during the semester and were completed as part of the course assignments. Participants used the learning texts provided in the course as basis for these argumentations.

### 2.4 Procedure

**Figure 3:**
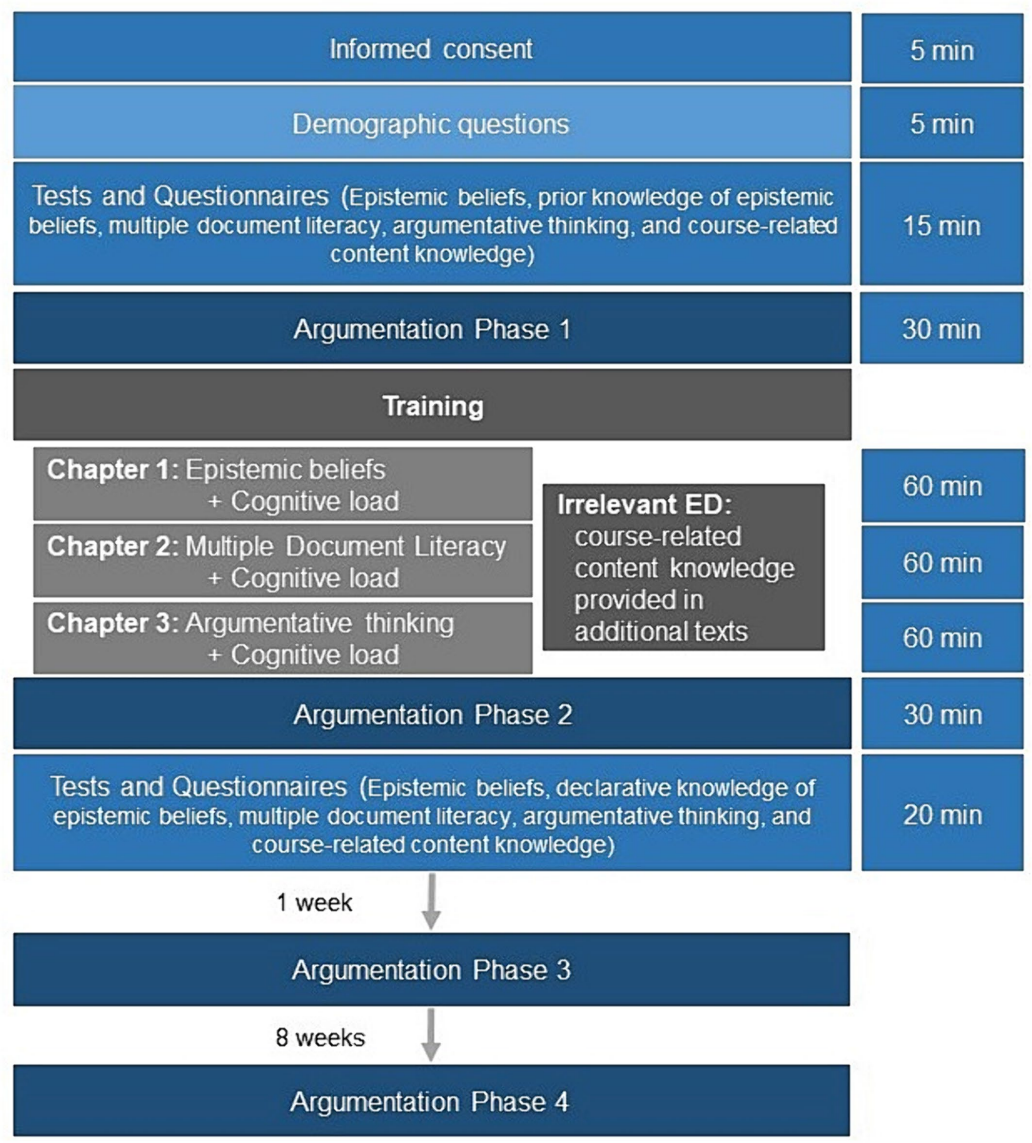
Overview of the procedure during the training session. ED = exemplifying domain.

Students were given information about the training and its components before giving informed consent. They were asked to answer demographic questions, before filling in the epistemic beliefs’ questionnaire (see Figure 3 for an overview and expected time needed). Students answered prior knowledge questions and wrote an argumentation about gender segregation in schools after reading three short texts about this topic (first argumentation; see Supplementary material E for excerpts of the argumentations). All participants were randomly allocated to conditions and started the three learning modules. After each module, participants were asked to indicate the cognitive load associated with the learning materials. Only the participants in the irrelevant examples condition were asked to read a text containing the relevant course-related content knowledge that the other group had received as exemplifying domain after each module. This expository text contained the same content as included in the worked examples of the relevant content group. The text was not structured into different arguments or as coming from different sources (see Supplementary material F). Compared to the materials the relevant content group received, the text was not integrated into a conversation or interspersed with explanations about the skill component being shown. While participants in the irrelevant content group read these texts, the relevant content group did not receive an additional task and could start the next task. Immediately after the training, all participants were asked to read three texts about the legal status of home-schooling in Germany and to write an argumentation about this topic (second argumentation). They completed tests assessing their declarative knowledge about the skill components and the relevant, course-related content knowledge. Lastly, participants completed the epistemic beliefs questionnaire a second time before they were thanked for their participation.

Later in the course, the participants wrote two argumentations as course assignments, one a week after the two-week training period had ended, the other approximately 9 weeks after the training period, although the exact timing varied between individual participants (third and fourth argumentation). Participants gave additional consent for their course assignments to be analyzed for research purposes. For participants who had provided their consent, the argumentations were included in the data analysis to test delayed training effects. Participants had no other practice opportunities during the course and did not receive additional instruction on writing argumentations.

### 2.5 Scoring

The argumentations were coded based on Toulmin (1958) argument schema and Kuhn’s argumentation structure (Kuhn, 1991). Answers received points for various components and the quality of their implementation (see Table 1). The quality of arguments, evidence, and conclusion was coded on a scale from *low quality* (0) to *high quality* (3). The presence of other indicators of argumentation quality, such as references to empirical data, was coded on a scale from *absent* (0) to *at least three present* (3). All scores were added up to form an overall indicator of argument quality.

**Table 1:** Coding schema for argumentations.

Aspect	Minimal points			Maximal points
Quality of argument (How convincing/new/ inspiring is the claim?)	0 (no valid claim)	1 (claim, but not well supported)	2 (Valid claim, transferred from texts)	3 (valid claim, transformed from texts)
Quality of evidence	0 (no evidence)	1 (anecdotal evidence)	2 (empirical evidence)	3 (empirical + study explained)
Quality of conclusion (synthesis, own theory or alternative theory)	0 (no conclusion)	1 (reiteration of own theory)	2 (summary of presented arguments)	3 (summary and implication/own deduction)
Presence of other components (warrants, backing, modals, qualifiers, reference to empirical data or evidence)	0 (absent)	1 (one component present)	2 (two components present)	3 (at least three components present)

The argumentations were coded by the first author without knowledge of the participants’ condition. A student assistant was instructed in using the coding scheme by the first author and coded 20% of the argumentations. Where the two raters coded differently, a consensus validation was performed, meaning that the two raters discussed each issue until an agreement and final solution was reached. The two ratings reached an interrater reliability of ICC (consistency) = 0.692. This reliability is just of moderate size; the complexity of the topic and the coding schema could have contributed to the differences between raters.

For one of the three universities, study regulations specified that students should write only one of the two delayed argumentations, and could choose which one to write. For students from this location, only three argumentations were included in the analysis.

## 3 Results

This study looked at whether (1) there was a main effect of training (participants showing more advanced epistemic beliefs and higher argumentation quality after the training than before), and (2) whether there are significant group differences due to the training type (participants receiving irrelevant exemplifying domain examples differing from those receiving relevant exemplifying domain examples in their cognitive load, declarative knowledge measures, epistemic beliefs, and argumentation skills). We used mixed factorial ANOVAs to analyze the data, as we wanted to look for repeated measures, between-subjects, and interaction effects. The same analyses were used to answer both research questions. The results will be reported in order of the research questions and not in order of the analyses run.

We used an alpha level of 0.05 for all statistical analyses. As effect size measure we used partial η^2^, qualifying values of <0.06 as weak effects, values between 0.06 and 0.13 as medium effects and values >0.13 as large effects (Cohen, 1988). Missing data were excluded listwise.

While we assumed that relevant examples (and thus simultaneous learning goals) could impede learning, a support for the null hypotheses might show that this was not the case. Bayes factors (BF) were calculated for findings related to the research questions that did not reach significance, to test whether the null hypothesis was substantially more likely than the alternative hypothesis. Bayesian statistics can give a probability for the null hypothesis given the data, so the BF can give an indication whether the data likely reflect the null hypothesis, that is, there was no difference between groups or measurements. We used a conservative JZS prior, so the probability that the BF reveals insufficient evidence is higher than a false confirmation of the null hypothesis.

As the declarative knowledge post-tests for the three skill components and the course-related content knowledge showed a significant negative skew, they were reverse scored and log-transformed to reduce the influence of possible ceiling effects.

### 3.1 Preliminary analyses

The experimental groups were comparable with respect to the assessed learning prerequisites (see Table 2 for descriptive values). ANOVAs were used to test group differences before the training. The results indicate that there were no significant differences between the experimental groups in terms of age, Abitur grade, or prior knowledge of the skill components and of the course content (all *p*s ≥ 0.515). Participants’ time between starting and completing the training varied from three to 244 h, and these data were positively skewed (2.245). In order to ensure that group differences were not caused by different amounts of time spent on the training, we compared the times used between the groups. We first looked only at participants with a training duration of 12 h or less, to exclude participants who had taken longer breaks or segmented the training over several days. For these participants (*n* = 65), we did not find a significant difference between experimental groups (*p* = 0.597, BF_01_ = 4.647). Looking at all participants, there was also no significant difference between groups (*p* = 0.249, BF_01_ = 4.049), supported by Bayes factors indicating that the null hypothesis (no difference between groups) was at least four times more likely.

**Table 2:** Mean scores and standard deviations for the demographics, prior knowledge tests, and training duration.

Variable	Relevant examples	Irrelevant examples	F	*p*
Age	24.23 (6.30)	24.26 (6.60)	<0.01	0.984
Abitur grade	1.53 (0.57)	1.52 (0.63)	0.02	0.883
PK Epistemic beliefs	1.68 (2.46)	1.74 (2.13)	0.03	0.863
PK MDL	1.37 (1.77)	1.53 (1.99)	0.25	0.617
PK argumentative thinking	2.29 (1.65)	2.34 (1.53)	0.03	0.870
PK content knowledge	3.32 (2.27)	3.59 (2.64)	0.43	0.515
Training duration (min) - under 12 h	345.4 (137)	363.8 (140)	0.28	0.597
Training duration (min) - all	2603.9 (3394)	1988.2 (2946)	1.34	0.249

PK = prior knowledge.

### 3.2 Increases in epistemic beliefs and argumentation quality

**Figure 4:**
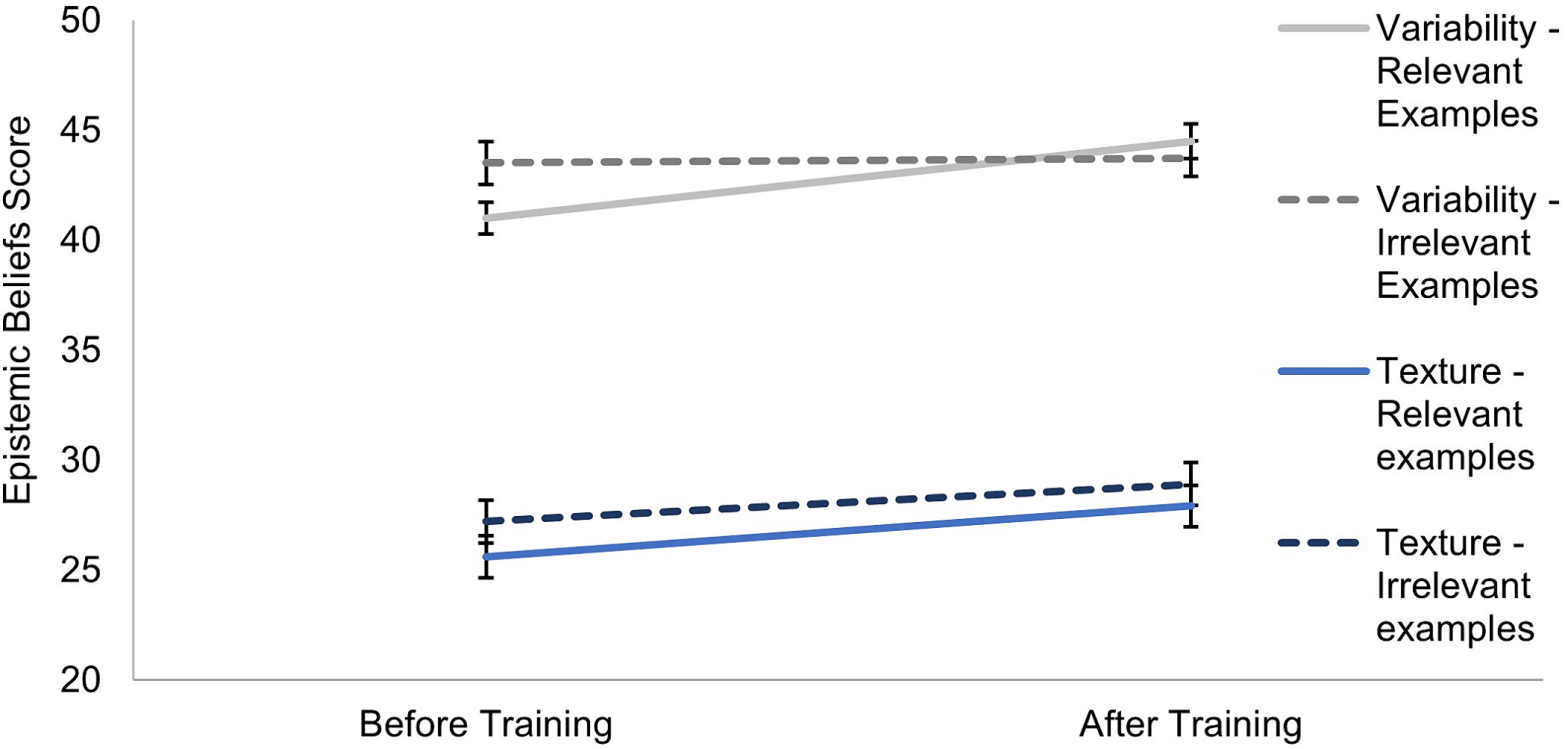
Means for the epistemic beliefs factors before and after training with standard error bars.

For the epistemic beliefs, we used mixed factorial ANOVAs to test for changes over time (H1), group differences, and an interaction between group and time (e.g., different changes over time in the two groups). Participants showed a significant increase in their epistemic beliefs scores after training (see Figure 4): The ANOVAs found significant differences for the two CAEB factors of Texture and Variability from before to after the training (see Table 3: Within columns). Although the descriptive values indicate only small changes in the scores, the effects across participants showed medium effect sizes (both η_p_^2^ ≥ 0.06).

**Table 3:** Results of mixed factorial ANOVA on epistemic beliefs factors before and after training.

Variable	RE		IR		Within			Between		
	Before	After	Before	After	*F*	*p*	η_p_^2^	*F*	*p*	BF_01_
Texture	25.6 (7.1)	27.9 (6.9)	27.2 (8.2)	28.9 (8.1)	11.2	0.001	0.084	1.09	0.299	5.45
Variability	41.0 (7.2)	44.5 (5.9)	43.5 (5.9)	43.7 (6.6)	13.9	<0.001	0.102	0.66	0.417	2.67^a^

RE = relevant examples; IR = irrelevant examples; mean scores shown with standard deviations in brackets; *n* = 124. *significant at α = 0.05 level.

a Bayes Factor < 3, indicates that evidence only anecdotal.(i.e., weak).

**Figure 5:**
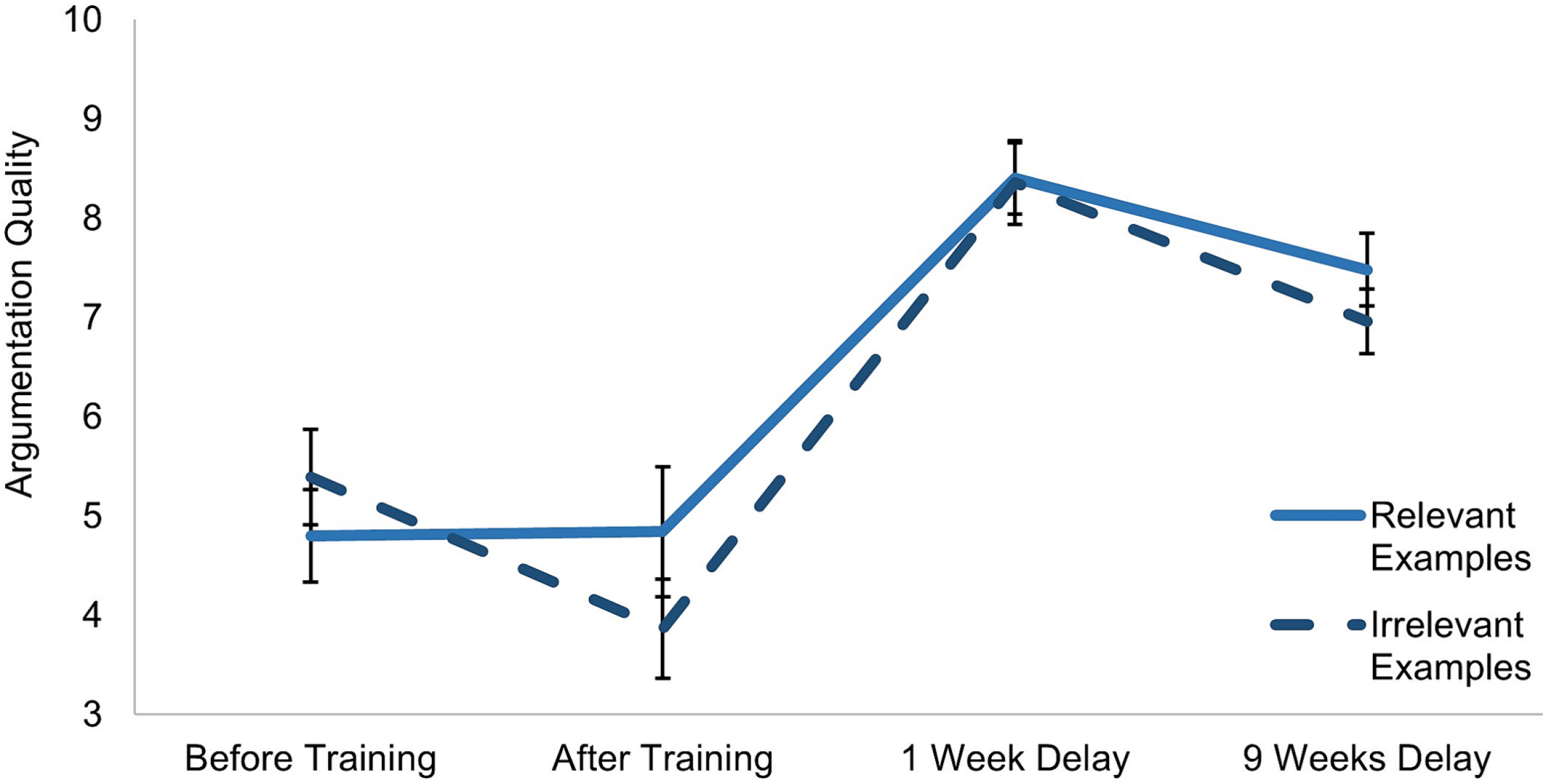
Mean scores and standard errors for the argumentation quality.

For the argumentation quality, a mixed factorial ANOVA with planned contrasts was used to compare the scores on the first argumentation to the following three argumentations, written immediately after, and approximately 1 week and 9 weeks after the training (H2). Surprisingly, participants showed a decrease in their argumentation quality scores from the first to the second argumentation (see Figure 5). The quality increased to the third and the fourth argumentation. The ANOVA indicated a significant measurement time effect on argumentation scores (*F*(3,153) = 40.07, *p* < 0.001, η^2^_p_ = 0.440). Planned contrasts showed significant differences between the first and second (*F*(1,51) = 6.67, *p* = 0.013, η^2^_p_ = 0.116), the first and third (*F*(1,51) = 57.71, *p* < 0.001, η^2^_p_ = 0.531) and the first and fourth argumentations (*F*(1,51) = 27.16, *p* < 0.001, η^2^_p_ = 0.347).

Our hypotheses were mostly confirmed, as participants showed higher epistemic beliefs scores after the training and higher argumentation quality for the delayed argumentations. Possible reasons for the decline in argumentation quality for the second argumentation will be elaborated in the discussion.

### 3.3 Group differences in cognitive load, declarative knowledge, epistemic beliefs and argumentation quality

We used between-subjects ANOVAs to test for group differences in the cognitive load and declarative knowledge measures (RQ1, RQ2). We tested whether having simultaneous learning goals would lead to higher intrinsic load, which could impact learning outcomes. However, participants receiving relevant examples did not differ from participants receiving irrelevant examples for any the cognitive load types. We found no significant differences for intrinsic, extraneous or germane load, further supported by BFs indicating that the null hypotheses were at least six times more likely, except for the germane load (see Table 4).

**Table 4:** Results of ANOVAs comparing relevant and irrelevant example groups on cognitive load types and declarative knowledge tests.

Variable	*n*	RE	IR	*F*	*p*	BF_01_
Intrinsic load	140	4.44 (1.19)	4.34 (1.09)	0.27	0.603	6.68
Germane load	140	4.88 (1.04)	4.60 (1.07)	2.48	0.117	2.34^b^
Extranous load	140	3.53 (1.11)	3.65 (1.16)	0.39	0.532	6.31
DK epistemic beliefs^a^	141	11.41 (2.86)	11.75 (2.29)	0.47	0.496	6.11
DK MDL^a^	141	12.81 (2.94)	13.53 (2.59)	5.11	0.025*	0.69
DK argumentative thinking^a^	141	11.62 (2.94)	11.75 (2.41)	0.001	0.974	7.63
DK content knowledge^a^	141	21.78 (6.80)	23.25 (5.53)	2.84	0.094	1.99^b^

RE, relevant examples; IR, irrelevant examples; DK, Declarative Knowledge.*significant at α = 0.05 level.

a Scores reversed and log-transformed for analysis.

b Bayes Factor < 3, indicates that evidence only anecdotal.

We also tested whether having simultaneous learning goals would affect the declarative knowledge of skill components and course-related content acquired, using the transformed scores for the declarative knowledge tests. Participants receiving relevant examples did not differ from participants receiving irrelevant examples for most of the declarative knowledge tests, except for knowledge about MDL. We found no significant differences between the two groups for the declarative knowledge tests about epistemic beliefs, argumentative thinking, or course-related content (all *p*s ≥ 0.094, see Table 4). The lack of group differences was further supported by the BFs indicating the null hypotheses were at least six times more likely for the declarative knowledge about epistemic beliefs and argumentative thinking skills (all BFs_01_ > 6.114). However, the BFs yielded no evidence favoring any hypothesis for declarative knowledge about course-related content (BF_01_ < 3.00). We found a significant difference between groups for declarative knowledge about MDL, with the group receiving irrelevant examples scoring higher than the group receiving relevant examples.

The mixed factorial ANOVAs of epistemic beliefs scores were also used to test for group differences and interaction effects with measurement time (RQ3). Overall, participants receiving relevant examples did not differ in their epistemic beliefs from those receiving irrelevant examples. We found no differences between the two groups for the Texture or the Variability factor (see Table 3: Between columns). For main effects of the experimental group, the non-significant results for the Texture were supported by the BF, indicating that the null hypothesis was at least five times more likely, but not for the Variability, which yielded no evidence favoring any hypothesis. There was a significant interaction between measurement time and group for the Variability (*F*(1,122) = 11.34, *p* = 0.001, η^2^_p_ = 0.085). The group receiving irrelevant examples started with higher scores, but they hardly changed from before (*M* = 43.51, *SD* = 5.93) to after the training (*M* = 43.68, *SD* = 6.61). The group receiving relevant examples showed an increase in Variability scores from before (*M* = 41.00, *SD* = 7.21) to after the training (*M* = 44.47, *SD* = 5.94).

In the mixed factorial MANOVA of the argumentation scores, we tested whether participants who received relevant examples differed in their argumentation quality from participants who received irrelevant examples (RQ4). The results indicate that participants did not differ in argumentation quality scores between conditions, as we found no overall significant difference between conditions for argumentation scores across the four argumentations (*F*(1,51) = 0.36, *p* = 0.554, η^2^_p_ = 0.007). This finding was further supported by BFs indicating that the null hypothesis was at least six times more likely (all BFs_01_ ≥ 6.50), except for the fourth argumentation (BF_01_ = 1.66) showing there was only anecdotal evidence for the null hypothesis.

Hence, with respect to our second research question, we found no disadvantages for the condition with relevant examples compared to the condition who received irrelevant examples for any of the cognitive load types, declarative knowledge scores, or the two CAEB factors, except for the knowledge of MDL. Overall, results for our exploratory research questions suggest that participants who received two simultaneous learning goals did not show a clear disadvantage in their declarative knowledge or the quality of their delayed argumentations.

Lastly, we ran bivariate correlations between the cognitive load measures and the learning outcomes (the declarative knowledge measures and the three argumentations written after the training). There were no significant correlations with any of the learning outcomes (see Table 5), suggesting that the cognitive load may not have been a mediator of the training effects.

**Table 5:** Results of bivariate correlations between the cognitive load measures, declarative knowledge measures, epistemic belief measures and argumentation scores for the argumentations written after the training.

Variable	ICL	GCL	ECL	DK EB	DK MDL	DK ATS	DK CK	Arg. 2	Arg. 3	Arg. 4	Texture
Germane load	0.73, <0.001										
Extraneous load	0.55, <0.001	0.49, <0.001									
DK epistemic beliefs^a^	0.02, 0.777	−0.12, 0.171	0.10, 0.244								
DK MDL^a^	0.10, 0.259	<0.01, 0.959	0.14, 0.111	0.53, <0.001							
DK argumentative thinking^a^	0.07, 0.391	−0.04, 0.674	0.13, 0.136	0.34, <0.001	0.48, <0.001						
DK content knowledge^a^	0.14, 0.094	0.04, 0.633	0.13, 0.125	0.52, <0.001	0.59, <0.001	0.50, <0.001					
Argumentation 2	<0.01, 0.959	−0.06, 0.507	−0.04, 0.619	−0.11, 0.213	−0.22, 0.013	−0.11, 0.219	−0.19, 0.030				
Argumentation 3	0.06, 0.512	−0.01, 0.921	−0.07, 0.459	−0.18, 0.068	−0.17, 0.075	−0.10, 0.309	−0.13, 0.194	−0.02, 0.902			
Argumentation 4	0.11, 0.358	0.20, 0.097	0.04, 0.761	0.09, 0.478	0.09, 0.495	0.12, 0.331	−0.07, 0.606	0.11, 0.413	−0.02, 0.875		
Texture	−0.11, 0.219	−0.17, 0.058	−0.15, 0.092	−0.04, 0.681	−0.08, 0.357	−0.08, 0.390	−0.08, 0.365	−0.03, 0.735	0.15, 0.135	−0.09, 0.492	
Variability	−0.15, 0.104	−0.11, 0.234	−0.13, 0.147	−0.23, 0.009	−0.14, 0.122	−0.05, 0.613	−0.21, 0.018	0.18, 0.051	0.14, 0.161	0.09, 0.508	0.41, <0.001

r-values and p-values for all correlations, pairwise exclusion. ICL, Intrinsic load; GCL, Germane load; ECL, Extraneous load; DK, Declarative Knowledge; EB, Epistemic beliefs; MDL, Multiple Document Literacy; ATS, Argumentative Thinking Skills; CK, Content knowledge; Arg., Argumentation.

a Scores reversed and log-transformed for analysis.

## 4 Discussion

We examined the effects of a relevant exemplifying domain incorporated in worked examples fostering cognitive skills or, more specifically, how simultaneous learning goals affected cognitive load and learning outcomes for both exemplifying domain and skill. Our hypotheses tapped on training effects on epistemic beliefs and argumentation scores. Our exploratory research questions concerned the effect of simultaneous learning goals by using a relevant exemplifying domain (course-related content knowledge) in the worked examples.

### 4.1 Training effects on skill development

Students showed a significant increase in their Texture and Variability beliefs before to after the training, partly replicating the results of Udvardi-Lakos et al. (2023), who found only an increase in the Texture beliefs. As epistemic beliefs usually develop slowly over the course of education, through experiences challenging existing beliefs and prompting the adjustment of prior assumptions (Baxter Magolda, 1996, 2004), this development is promising.

We also examined short- and long-term effects of our training using immediate and delayed argumentations. The training seems to have led to significantly increased quality of the third and fourth argumentations. Although we had no condition to control for effects of practice or changes in motivation on argumentation quality, this increase in quality could suggest an increase in students’ skills. Surprisingly, the argumentation written immediately after the training was not better than the argumentation before the training.

There are several possible reasons for these findings. One reason could be a reduced motivation after going through the long training, whereas the two delayed argumentations were written as part of the course requirements, so students could have felt more extrinsically motivated to perform well on these tasks.

Possibly, the training could have had a delayed effect on skill improvement, as students may have needed some time to consolidate what they had learnt. That is, students could have shown a utilization deficiency, where they can use a skill, but show little or no gain in task performance as the new skill requires (too) many cognitive resources (Clerc and Miller, 2013; Miller, 1994). In our case, students may have understood the components involved in argumentative thinking skills, but could not apply this understanding efficiently, as executing the skill possibly required too many resources when transferred to a new topic application (Clerc and Miller, 2013; Miller, 1994). In addition, as the second argumentation was written immediately after the training, students could have depleted their cognitive resources and could have had less capacity to use their argumentative thinking skills to write a good argumentation. Students reported that the training took longer than expected, with some students spending up to 8 h on the materials. Cognitive exhaustion could also explain a utilization deficiency: If students had depleted their cognitive resources, they may not have had enough resources left to apply their argumentative thinking skills effectively (Clerc et al., 2021). In the delayed argumentations, students had time to recover from the exertion of the training and thus been able to apply their understanding efficiently, leading to improvements in their argumentation quality.

The influence of cognitive exhaustion on argumentation quality could also explain why the irrelevant exemplifying domain group appears to have performed worse than the relevant exemplifying domain group: The descriptive data seem to indicate that the relevant exemplifying domain group did not change in their argumentation quality from before to immediately after the training, while the irrelevant exemplifying domain group did decrease. Students in the irrelevant exemplifying domain group had to read additional texts containing the course-relevant content. This task could have exerted additional demands on students’ cognitive resources, leaving them more fatigued than the group with the relevant content integrated in the worked examples. While students did not have to process two learning goals at the same time, they did have to use additional resources to process the texts containing the course-relevant content. This difference could explain why the two groups seem to differ in their argumentation quality right after the training. However, an exploratory analysis suggests there was no significant interaction between the groups and time from the argumentation written before to the one written immediately after the training (*F*(1,125) = 1.52, *p* = 0.229).

### 4.2 Comparison of relevant and irrelevant content in worked examples

We assumed that simultaneous learning goals might lead to higher cognitive load and thus lower students’ learning outcomes for declarative knowledge of skill components, course-related content knowledge, epistemic beliefs, and argumentation quality. However, when comparing the group that received the relevant exemplifying domain to the group receiving the irrelevant exemplifying domain, no significant differences were found for any of the cognitive load types.

This finding was unexpected, as CLT suggests that including a relevant exemplifying domain would increase the intrinsic load due to the increased number of elements that need to be processed. In previous studies on skill acquisition, simple and familiar exemplifying domains were used to avoid high cognitive load demands. Based on the effort regulation framework, learners could actively invest more effort into content perceived as relevant for their goals (de Bruin et al., 2020). This mechanism could also mean that learners report a higher germane load as they engage in more learning activities to achieve the two simultaneous learning goals, for example, by elaborating the exemplifying domain content. However, both higher intrinsic and germane load would depend on the learners perceiving the exemplifying domain as relevant to their learning goals. Course-related content knowledge would fit learners’ goals of passing the university course, so learners could invest more effort into processing worked examples with a relevant exemplifying domain. In comparison, irrelevant exemplifying domains would demand little extraneous load, as the contents would not need to be processed elaborately, and learners would be unlikely to invest effort into content knowledge not relevant for their goals. However, learners did not perceive significant differences in intrinsic, extraneous or germane load.

These findings could be interpreted in different ways. First, previous studies used simple, familiar exemplifying domains not relevant for learners’ goals and assumed that these contents would exert little extraneous load. This assumption may be false, as our groups reported equal levels of extraneous load, so irrelevant topics might also induce high extraneous load that hinders learning. In comparison, the relevant exemplifying domain used in this study did not increase load further. Second, learners in both groups may have actively processed the contents of their respective exemplifying domains and thus invested equal levels of effort. As we did not assess perceived relevance, we cannot rule out this explanation. Third, the cognitive demands of our relevant exemplifying domain may have been comparatively low, as the topics covered in the course are related to topics of other psychology courses, so learners may have had significant prior knowledge. The prior knowledge could have helped them process the contents of the relevant exemplifying domain more effectively, thereby not increasing the intrinsic load compared to the irrelevant exemplifying domain.

These interpretations deserve further research; the assumptions of CLT and the distinction of extraneous and intrinsic cognitive load and their roles for learning seem unclear in this context. Whether an exemplifying domain is seen as extraneous or intrinsic load could depend not only on instructional design but also on learners’ goals, and instructors need to consider learners’ motivation to ensure that they design their instructional materials so learners focus on aspects relevant for their learning outcomes. Furthermore, we could not distinguish between the load demanded by the worked examples, and the effort learners were willing to invest based on the relevance of the exemplifying domain and their learning goals. Future projects could include measures that differentiate between the effort demanded by the learning materials (cognitive load) and the effort actively invested by learners (Klepsch and Seufert, 2021).

Nevertheless, our results suggest that students were not cognitively overwhelmed by two simultaneous learning goals. We found no significant differences between the relevant and irrelevant conditions for the declarative knowledge about epistemic beliefs, argumentative thinking skills, or course-related content knowledge. Although we did not use comparable tests for the prior knowledge and declarative knowledge after the training (as the post-test items would have been too difficult before the training and could have demotivated learners) the descriptive results suggest that participants increased their knowledge of the skill components and the course-related content knowledge.

The two groups did not differ on the two delayed argumentations either. We can make the tentative assumption that there were no group differences, that is, students did not struggle with the learning materials when they were given two simultaneous learning goals. Despite the assumptions of CLT, a higher number of (relevant) elements did not increase the perceived intrinsic load and did not impede skill acquisition.

We found significant interactions between the measurement time and the groups for the epistemic beliefs. While students who received relevant examples showed a significant increase in advanced beliefs relating to the variability of knowledge, students who received irrelevant examples did not show such an improvement. However, since students who received irrelevant examples showed more advanced beliefs before the training than students who received relevant examples, their beliefs may have been harder to change to even more advanced views.

Overall, our findings suggest that having two simultaneous learning goals, asking students to learn about both skill components and content knowledge, does not impede skill development. Incorporating relevant content knowledge into worked examples does not seem to increase the difficulty of the learning materials, as CLT would have suggested, explaining why students showed hardly any differences in the knowledge and skills measures. These finding confirm the assumption of Wecker et al. (2016) that incorporating content knowledge into the teaching of different skills could help make trainings more efficient.

### 4.3 Implications for teaching

When designing learning materials for students, it thus seems possible to integrate declarative content knowledge relevant for learners’ learning goals into worked examples used to foster skill acquisition. This approach could increase efficiency in many learning contexts where learners have to acquire both skills and content knowledge in limited time. It could also increase learners’ motivation to study the worked examples: If learners see the relevance of the topic being covered, the relevance could increase the utility value associated with the learning materials, and thus, learners’ motivation to engage with the learning materials (Eccles and Wigfield, 2002; Wigfield and Eccles, 2000). Worked examples with relevant content also increase the authenticity of the task, as learners are taught in a context similar to the contexts where they should be able to apply the skills by themselves later. Furthermore, learning about skill application using relevant content could foster a deeper understanding of the content—if learners have to understand the arguments about a specific topic, they may gain a better understanding of the different aspects and their interrelationships than they would from reading an expository text instead. Relevant content integrated in worked examples could thus foster efficiency and learning outcomes for skill and content.

However, it is also possible that learners get distracted from the learning goal of acquiring the skill when they only focus on the relevant content and do not realize that two learning goals are being addressed by the same materials. Clear instruction concerning the relevance of the content and the skill should be given, so learners know where to invest effort and where to ignore contents not relevant for learning goals (Eitel et al., 2020; McCrudden and Schraw, 2007). By providing worked examples that include relevant content, learners could also be led to copy the examples rather than really engage with a task when using the skill on their own (e.g., writing an argumentation with the same main claims as given in an example to avoid making a mistake). Learners may perceive the provided examples as ideal solutions rather than options for solving a task, especially given the ill-structured nature of heuristic skills. Learners may have trouble distinguishing between aspects of skill acquisition (showing the use of specific principles or heuristic strategies), and aspects of the content knowledge, which may have right or wrong answers, or strong and weak claims (e.g., there is currently more evidence to suggest that home-schooling can be detrimental to learning than there is for the opposite claim). In order to address these issues, instructors could use multiple examples from the same general domain, as used here, but different enough so that by comparing the examples, learners can identify main principles of the skill as applied to different topics (Renkl, 2023).

### 4.4 Limitations and future research

A limitation of our design is that students’ perceived relevance of the exemplifying domains was not assessed. The students received information that course-relevant content would be used during the training, but they were not told specifically which content was considered as course-relevant. As the students could have had a different understanding of which topics are relevant to them compared to what we saw as relevant for course performance, it is possible that students invested equal amounts of effort into “irrelevant” exemplifying domains than they did for “relevant” exemplifying domains. This difference in understanding of the relevance of topics could have led to the lack of group differences. In future studies, the perceived relevance of students should be assessed, or a clear instruction could be given concerning the relevance of specific topics for students’ learning goals.

Additionally, we did not measure motivation, for example achievement goals or engagement, of the students and thus the results could be influenced by differences in how important performing well in the course was for students. Generally, German psychology students experience a high external pressure to perform well in undergraduate degrees, due to the much lower number of spaces in the Master programs that lead to the psychotherapy training that many students aim for compared to the number of students who finish the undergraduate degree. Acceptance to the Master program is mainly based on undergraduate grades, so performing well in all courses is seen as highly relevant by most students. However, we do not know whether this was the case for all students in our sample. The achievement goals could influence students’ internal motivation and could play a considerable role with respect to instructional effects (Ames and Archer, 1988; Grant and Dweck, 2003). As we did not measure motivation and overall engagement, these factors could influence learning outcomes and should be included as possible moderators in future studies.

We found no significant associations between the cognitive load measures and the learning outcome measures, although we assumed that the cognitive load would determine the learning outcomes. As such, our findings have to be interpreted with caution, as the relationship between cognitive load reported by the students and their learning outcomes does not fit the assumptions of cognitive load theory (Sweller et al., 1998, 2019) or the effort monitoring and regulation framework (de Bruin et al., 2020). One issue could be that cognitive load was assessed by self-report items. Hence, future studies should include more objective measures of cognitive load and effort invested into learning.

The two delayed argumentations were also assessed as part of the course requirements; thus, the relevance of these argumentations was higher than for the first two argumentations. While we ensured that the amount of information and length required for the different argumentations were the same as for the pretest and immediate posttest argumentations, and the argumentations were graded as pass/fail, students could have felt more pressure to do well in the delayed argumentations. However, due to the long duration of the experiment and the effort needed for the argumentations, we avoided the risk of a high level of attrition by providing some incentive to complete the delayed argumentations. Additionally, the order of the topics of the argumentations was not counterbalanced, so topic-related effects, such as topic difficulty or familiarity, could have influenced the argumentation quality as well.

Furthermore, students could spend as much time on the training as they wanted to, and they could take breaks to avoid cognitive exhaustion. It is unclear whether receiving two simultaneous learning goals meant that students with relevant examples spent more time on the training, although our rough measure of training duration indicated that for students who completed the training in a single day, there were no differences between those who received relevant examples and those who received irrelevant examples. As the participants in the irrelevant content group had to read three additional texts with the course-relevant knowledge, this finding could indicate that studying worked examples with relevant content requires more time than irrelevant content. For future studies, the time-on-task in different learning phases or tasks should be measured to assess efficiency of the two groups.

The training used in the current study took place online, asynchronously, with learners deciding when to participate and how many breaks to take. As the course in which the training was implemented used a hybrid format with several online, asynchronous learning sessions, the training fit the overall instructional design used. However, this format also poses issues in relation to adherence to instructions and the engagement with the learning activities (e.g., Hollis and Was, 2016; Puzziferro, 2008; Tratnik et al., 2019), such as elaborating the strategies shown in the worked examples. The time invested into learning could vary widely between participants in this format, affecting learning outcomes. Additionally, learners did not have the opportunity to ask for support, to receive feedback, or ask questions. Conceivably, providing the training synchronously online or even in a face-to-face format could affect learning outcomes by aligning time-on-task between learners, increasing engagement with and adherence to the learning activities, and providing opportunities for exchange (Kemp and Grieve, 2014). While some studies suggest no differences between online and face-to-face teaching formats (Butts et al., 2013; Mulaimović et al., 2024), future studies should implement a more controlled environment for learning to ensure that these aspects actually do not influence the pattern of findings.

Another question is whether the skills could transfer to a new knowledge domain. In our study, the skills were taught and assessed using different topics from the “Pedagogy for Psychotherapists” course. As these topics belonged to the same sub-domain of psychology, it would be interesting to test in a future study whether students could use the newly developed skills when researching a different domain. The finding that the control group did not differ in learning outcomes although students received content knowledge from different domains suggests that the skills should transfer to new domains. However, as students were assessed using examples from the course content, another task to assess transfer would be helpful in future studies.

Overall, our study was a field study conducted in a regular psychology course, and as such, we could not control many variables that could have impacted cognitive load and argumentation quality, such as topic-related effects. In the future, we plan to conduct a more controlled study in a laboratory setting, to control for these influences and hopefully replicate our results.

## 5 Conclusion

Our study suggests that the training could have fostered advanced epistemic beliefs, MDL, argumentative thinking skills, and content knowledge. The training seems to have had a positive effect on students’ skills even 9 weeks after the experimental session. Our findings indicate that relevant content knowledge can be integrated into worked examples and used as additional learning goal for learners without increasing cognitive load or impeding students’ skill development. Overall, worked examples are a very promising method for teaching both content knowledge and complex skills.

## Data availability statement

The datasets presented in this study can be found in online repositories. The names of the repository/repositories and accession number(s) can be found at: https://osf.io/guw6n/?view_only=9bfedaaf05a64bb7a13e8d274c1bdbd8, Open Science Framework.

## Ethics statement

The studies involving humans were approved by the ethics committee of the University of Freiburg (approval code 274/20). The studies were conducted in accordance with the local legislation and institutional requirements. The participants provided their written informed consent to participate in this study.

## Author contributions

NU-L: Writing – original draft, Methodology, Formal analysis, Data curation, Conceptualization. MW: Writing – review & editing, Resources. JA: Writing – review & editing, Resources, Funding acquisition. AR: Writing – review & editing, Supervision, Conceptualization.
