# Supplementary material for: Example-based learning in heuristic domains: can using relevant content knowledge support the effective allocation of intrinsic, extraneous, and germane cognitive load?
Source: Front Psychol. 2024 Sep 23;15:1387095. doi: 10.3389/fpsyg.2024.1387095 (PMC11457169; doi:10.3389/fpsyg.2024.1387095)
Supplement: Supplementary file 1 [file Table_1.DOCX]

Supplementary Material A

**Supplementary Table A.** Excerpts from the Modules in the Relevant Exemplifying Domain Condition

| Learning Goals | 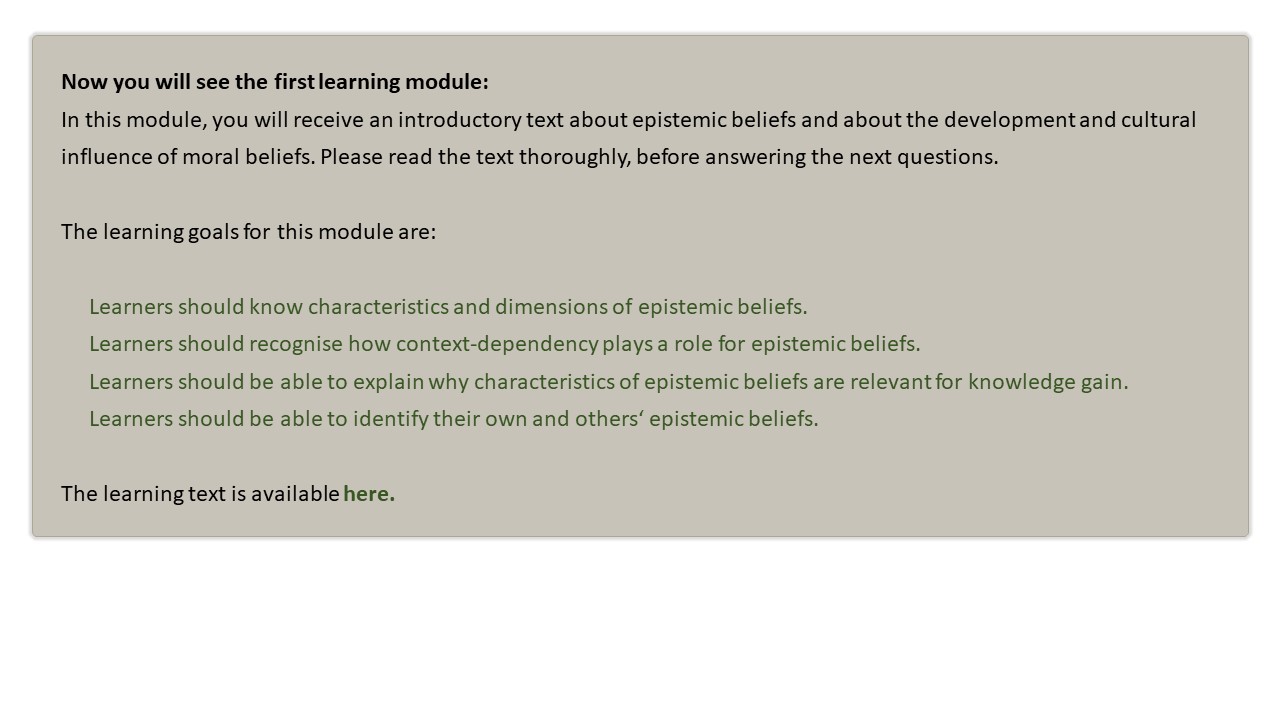 |
| --- | --- |
| Introductory Text | 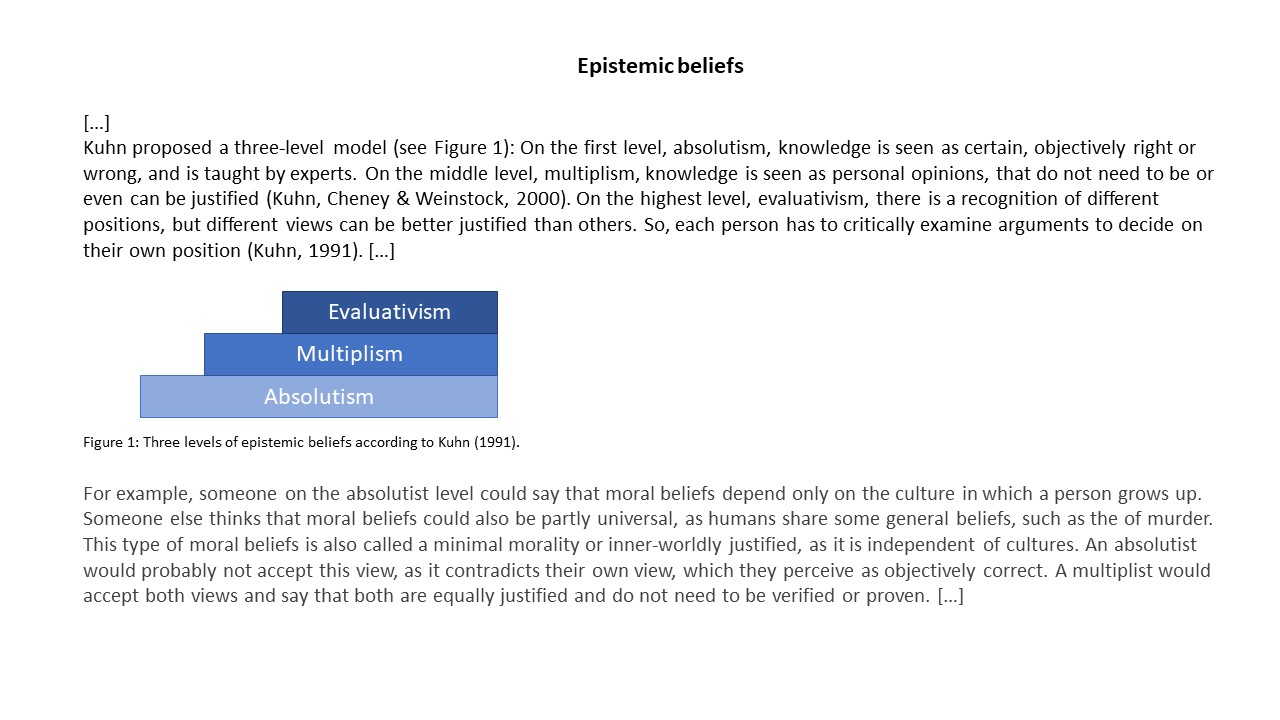 |
| Screen-shot of Example-Based Video About Epistemic Beliefs | 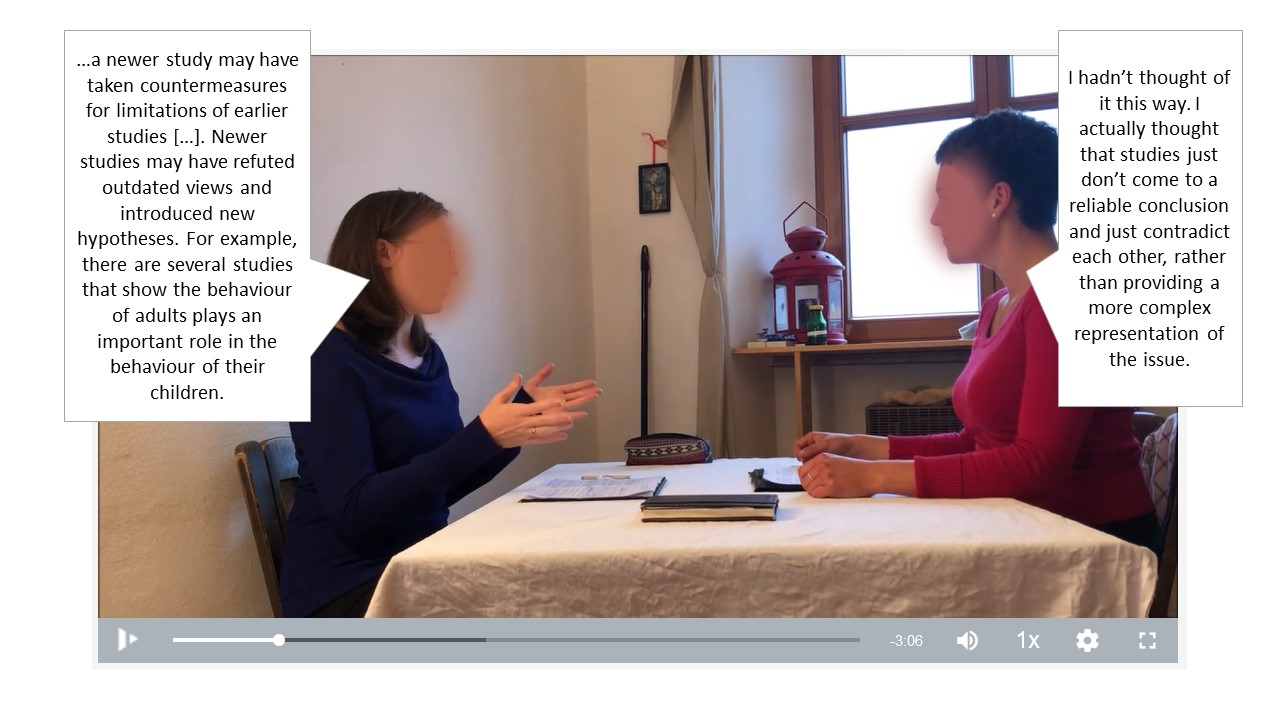 |
| Self-Explanation Prompts | 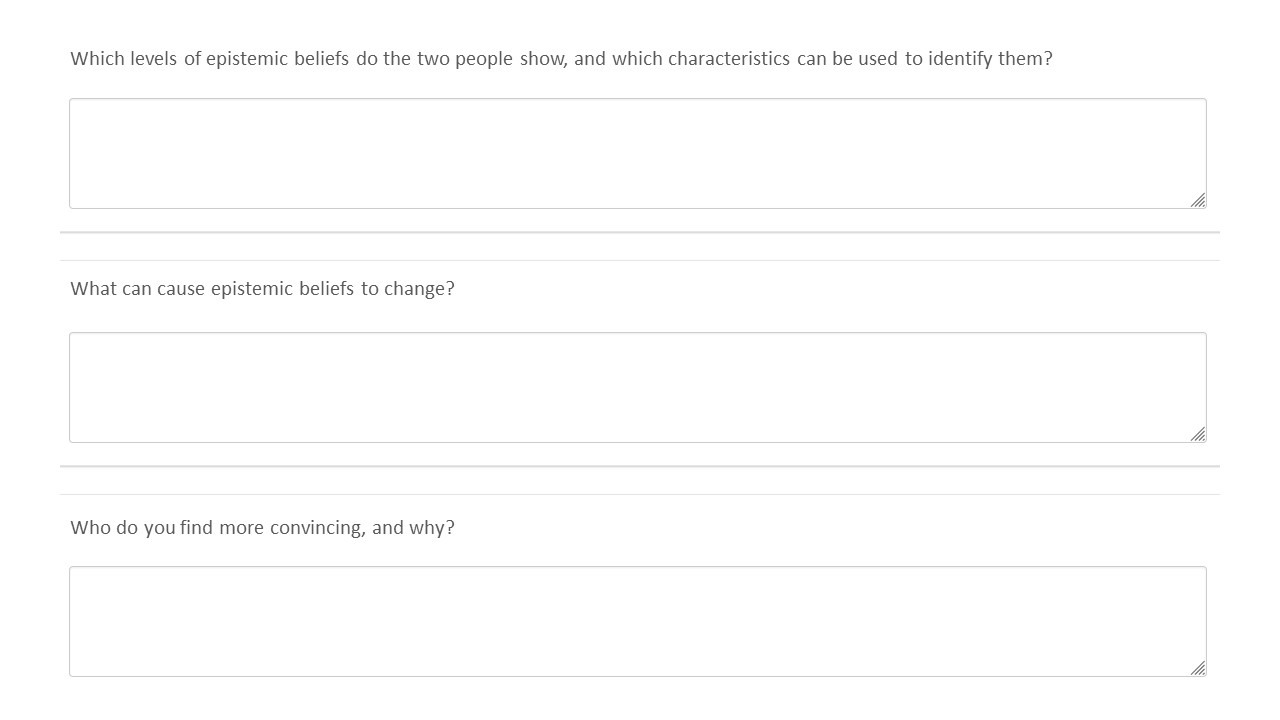 |
| Screen-shot of Example-Based Video About Argumentative Thinking | 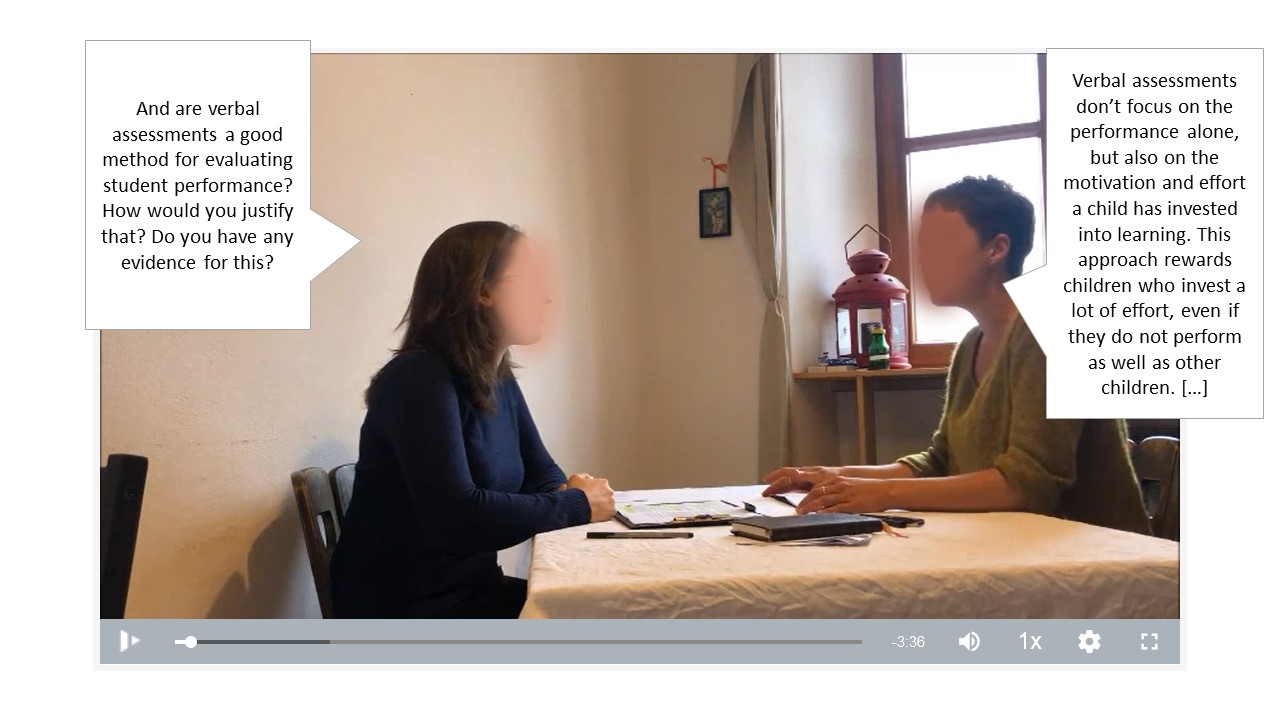 |
| Self-Explanation Prompts | 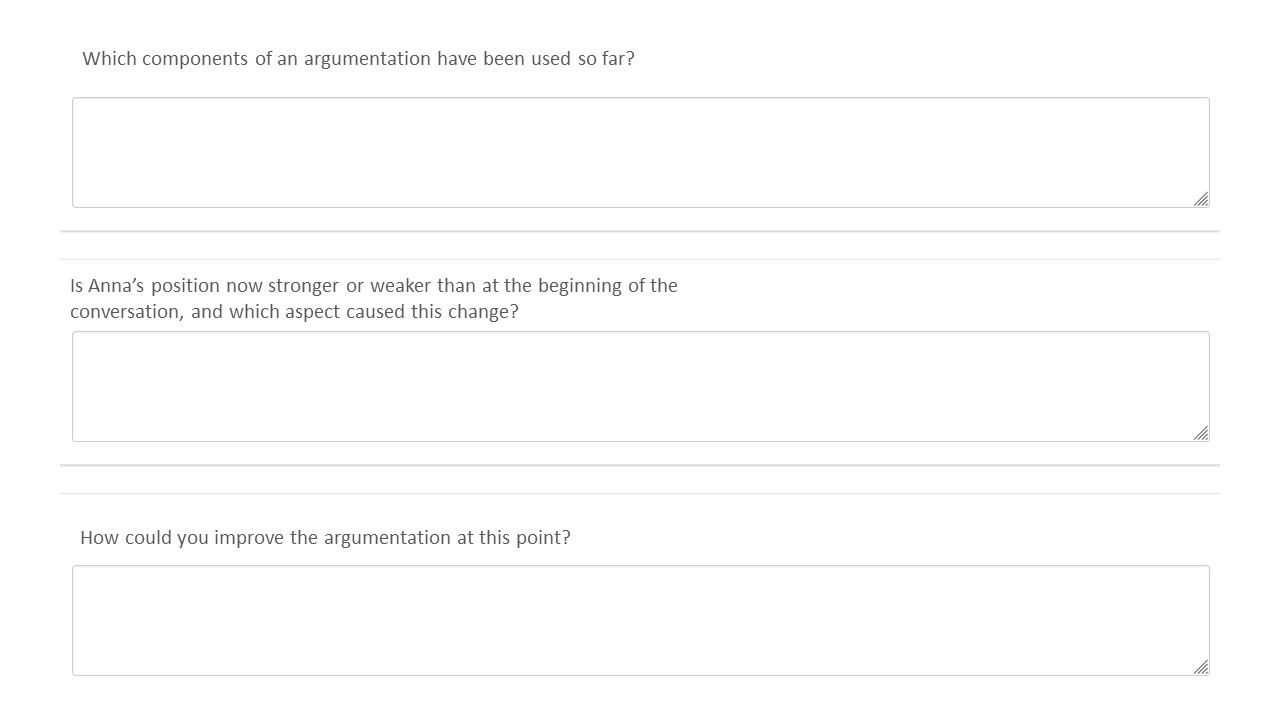 |

*Note*. All content was translated from German by the author. Speech bubbles were added to the video screenshots to show what the characters were discussing in the videos.

**
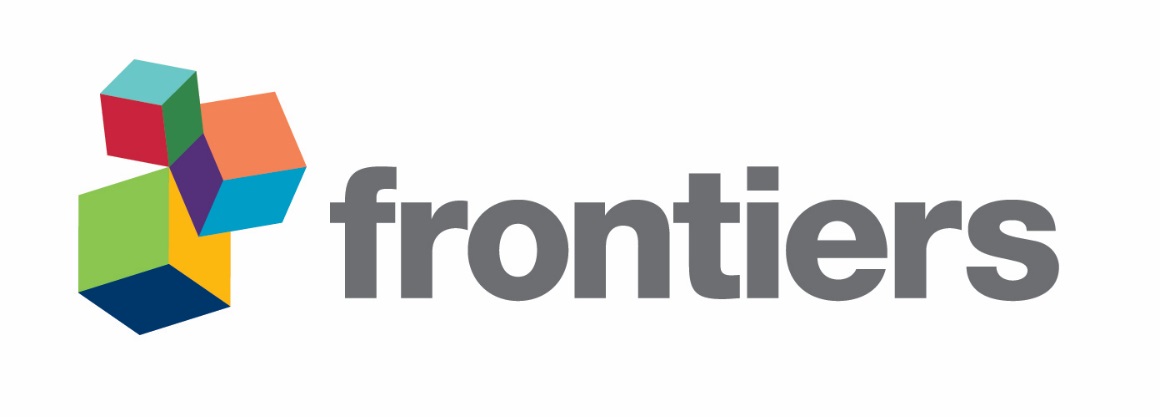
**
