# Supplementary material for: Example-based learning in heuristic domains: can using relevant content knowledge support the effective allocation of intrinsic, extraneous, and germane cognitive load?
Source: Front Psychol. 2024 Sep 23;15:1387095. doi: 10.3389/fpsyg.2024.1387095 (PMC11457169; doi:10.3389/fpsyg.2024.1387095)
Supplement: Supplementary file 2 [file Table_2.DOCX]

Supplementary Material B

**Supplementary Table B.** Measures and Their Reliabilities

| Concept | Measure | Reliability (Cronbach’s alpha) |
| --- | --- | --- |
| Epistemic beliefs  Pre-test  Texture  Variability  Post-test  Texture  Variability | CAEB (Stahl & Bromme, 2007) | .805  .747  .798  .798 |
| Cognitive load  Intrinsic load  Germane load  Extraneous load | 2-3 items each (Klepsch et al. 2020) | .859  .799  .849 |
| Declarative knowledge post-test  Epistemic beliefs  MDL  Argumentative thinking  Content knowledge | 15 items  30 items | .957  .976  .974  .978 |

**
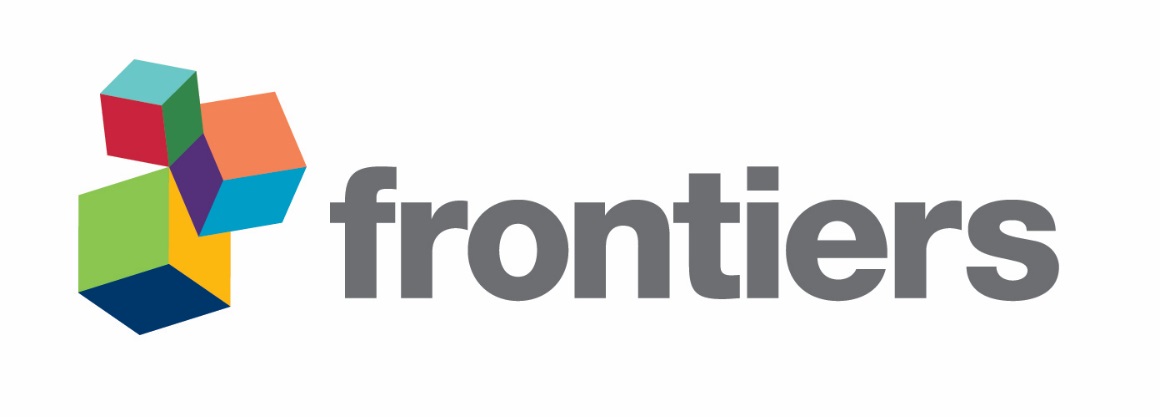
**
