# Supplementary material for: Example-based learning in heuristic domains: can using relevant content knowledge support the effective allocation of intrinsic, extraneous, and germane cognitive load?
Source: Front Psychol. 2024 Sep 23;15:1387095. doi: 10.3389/fpsyg.2024.1387095 (PMC11457169; doi:10.3389/fpsyg.2024.1387095)
Supplement: Supplementary file 3 [file Table_3.DOCX]

Supplementary Material C

**Supplementary Table C.** Items from the Prior Knowledge Tests

| Concept | Items |
| --- | --- |
| Epistemic beliefs | Do you know the concept of “epistemic beliefs”? |
|  | Please name the three levels of epistemic beliefs according to Kuhn. |
|  | Do you know the dimension of “certainty of knowledge”? If yes, please characterize this dimension in 1-2 sentences. |
| Multiple Document Literacy | Do you know strategies to learn from multiple documents? |
|  | Which models are constructed when reading multiple documents? |
|  | Do you know the term “corroboration”? If yes, please explain this concept in relation to multiple documents in 1-2 sentences. |
| Argumentative thinking | Do you know the components of an argumentation? |
|  | Do you know what modals are? If yes, please describe these in 1-2 sentences. |
|  | What is a “synthesis” in the context of an argumentation? Please define this in 1-2 sentences. |
| Course-related content | Have you ever completed a course on developmental and/or pedagogical psychology? If yes, how many? |
|  | Please name five key points from the findings of the Hattie study (2009). |
|  | Do you know the concept of the “Nature-Nurture-Debate”? If yes, please describe this in 1-2 sentences. |

**
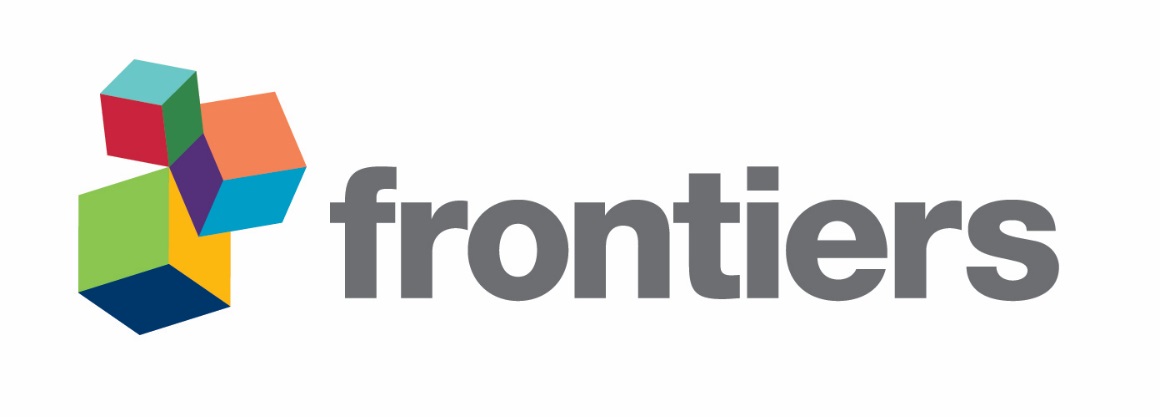
**
