# Supplementary material for: Example-based learning in heuristic domains: can using relevant content knowledge support the effective allocation of intrinsic, extraneous, and germane cognitive load?
Source: Front Psychol. 2024 Sep 23;15:1387095. doi: 10.3389/fpsyg.2024.1387095 (PMC11457169; doi:10.3389/fpsyg.2024.1387095)
Supplement: Supplementary file 4 [file Table_4.DOCX]

Supplementary Material D

**Supplementary Table D.** Example Items from the Declarative Knowledge Post-Tests

| Example Items About Epistemic Beliefs | | |
| --- | --- | --- |
| Several theories propose that epistemic beliefs develop throughout one’s life in a specific sequence. | **True** | False |
| Younger children are more likely to believe that they need to construct their own knowledge and should not listen to authorities. | True | **False** |
| Students who see the development of moral beliefs and the self-concept as independent topics may struggle more while learning than students who connect knowledge across several topics. | **True** | False |
| Example Items About MDL | | |
| In order to judge the usefulness of an information source, you should mostly consider the length of the text. | True | **False** |
| Contextualization is a strategy that can be used to consider possible biases in the views presented by an author. | **True** | False |
| I have found a newspaper article in which the author reports that most parents are against inclusive education. I would thus judge the verifiability of this information source as high. | True | **False** |
| Example Items About Argumentative Thinking | | |
| Argumentative thinking can help make connections between different pieces of information and thus supports learning outcomes. | **True** | False |
| If you formulate exceptions for your argument, you already admit that your position is not very strong. | True | **False** |
| The argument "Students should spend less time consuming media so they can focus more on school. Children who consume less media have more time for homework." is sufficiently substantiated. | True | **False** |
| Example Items About Course-Related Content | | |
| The parenting style and the acceptance of aggressive behaviour in the family can influence the relationship between violence-containing media and aggression in children. | **True** | False |
| The tracking system should lead to higher homogeneity in students’ learning potential in the different school types, but actually leads more to homogeneity in students’ eagerness to learn. | **True** | False |
| In order to motivate children, their work should not be compared to their previous performance, but with the performance of other class members. | True | **False** |

**
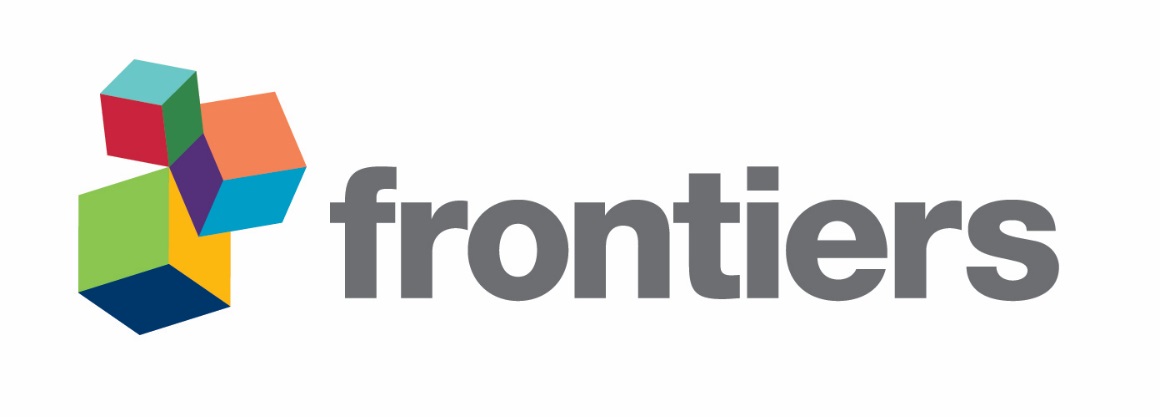
**
