# Supplementary material for: Example-based learning in heuristic domains: can using relevant content knowledge support the effective allocation of intrinsic, extraneous, and germane cognitive load?
Source: Front Psychol. 2024 Sep 23;15:1387095. doi: 10.3389/fpsyg.2024.1387095 (PMC11457169; doi:10.3389/fpsyg.2024.1387095)
Supplement: Supplementary file 5 [file Table_5.DOCX]

Supplementary Material E

Excerpts from the Argumentations

The following excerpts (ca. 190-250 words) have been taken from longer argumentations (ca. 400-1000 words) from the four measurement times: immediately before, immediately after, one week, and nine weeks after the intervention.

### Example 1: Argumentation About Gender Segregation in Schools (Before Intervention)

“On the one hand, the gender-specific separation seems to offer advantages. Boys and girls have different strengths in certain subjects, which can be strengthened or promoted more strongly. Boys are more interested in subjects in the natural sciences and could push girls into the background through their stronger commitment. By separating them by gender, girls could be more involved in such subjects. On the other hand, interaction with only one gender leads to a reinforcement of existing stereotypes. Children cannot make their own experiences in this regard and check the stereotypes for "correctness" due to the separation, which is, however, an important factor in reducing stereotypes (and especially in negative attitudes), as socio-psychological studies show. Ultimately, however, these arguments show little empirical power because, according to one meta-analysis, these effects were due to other variables and not just the separation of the sexes in the classroom. Moreover, there were no significant differences between segregated and co-ed instruction in terms of student achievement and interest. In contrast, it makes more sense to design lessons in a gender-sensitive manner and to address the interests of both genders. Fundamentally, it should be about student learning. And as Hattie (2009) has already shown, learning takes place in the classroom and is influenced more by teaching factors than by structural conditions. […]”.

### Example 2: Argumentation About the Legalization of Home-schooling (After Intervention)

“[…] Many parents also fear that a public-school education will not prepare children for the world of work. They argue that children benefit from individualization in home-schooling, that strengths and weaknesses can be better taken into account, and that exaggerated ideas of achievement can be avoided. In addition, researchers of constructivist approaches assume that children learn best when they can follow their interests and become active themselves (Holt, 1984). Studies show that home-schooling children make greater learning gains (Cheng et al., 2016), perform better on academic tests (Clark, 1994), have good self-esteem, and are socially and emotionally adjusted. Home-schooling is attractive from a governmental perspective because it saves financial and material resources for children who are home-schooled. Critics, however, point out that school should serve the socialization and equal opportunities of all children, as well as strengthen cohesion among citizens and prevent child labour. They fear that home-schooling parents view academic education as secondary, focus on their norms about religion and morality, and teach a limited worldview, not allowing their children to critically engage with that worldview. Critics suggest that these children learn less self-determination and important skills such as debating, that they may have limited access to information and knowledge content. Children from home-schooling are also not familiar enough with specifications in the education system and may have difficulty transitioning into training or college. […]”

### Example 3: Argumentation About Approaches to Gifted Education (One Week After Intervention)

“[…] The need to support gifted and talented children (Rogers, 2007) is obvious: gifted children, just like other students, have the right to experience success by mastering challenges at school. Such an experience of competence is necessary to recognize one's own abilities (Deci et al., 1994) and to develop motivation to improve those abilities. Now, it could be argued that the experience of competence is sufficiently fostered when gifted students recognize that they are much more capable of solving tasks than their classmates. However, studies have shown that this kind of affirmation is not sufficient: children who cannot fully exploit their performance capacities suffer from underchallenge and loss of motivation, which can have a negative impact on their mental health (Preckel et al., 2012). The promotion of the experience of competence to protect motivation by teachers in the sense of individual adjustment of the learning level and pace to the respective students is therefore advisable (Weinert, 1997; Wild, 2001). Based on the above considerations, it seems obvious to prefer segregation to integration in order to avoid a reduction of motivation with the help of competence-based support for highly gifted children. […]”

### Example 4: Argumentation About Digitalization in Schools (Nine Weeks After Intervention)

“[…] In schools, digital media represent new forms of information and interaction, further opportunities for individual teaching, and diversity in the instruction of students. For this to happen, however, a certain infrastructure, teaching materials and concepts must first be available at the schools, and the teachers should have professional competencies in the area of digital media so that they can be used optimally. On the one hand, this holds potential for the teaching context, but on the other hand, it brings with it a number of challenges. Digital media have a great impact on the availability of information. The Internet offers a large amount of information that can be accessed in a short time with the help of search functions. Lessons can be enriched by this and also for the follow-up of the learners this represents extended possibilities (Gerjets & Scheiter, 2019). To be able to use this flood of information, the learner needs content-specific prior knowledge, must be able to control him/herself, and must have beliefs that are differentiated and epistemic. If these are available, the learner can relate the information from the various sources and the associated different perspectives to each other. Optimally, this results in a differentiated mental representation of the information (Gerjets & Scheiter, 2019). A correct assessment and classification of the many different sources is an important competence here. […]”

**
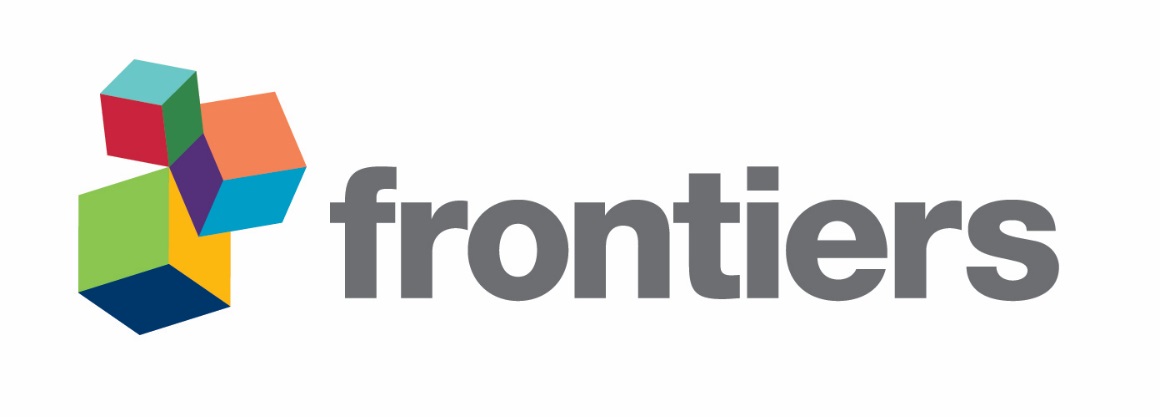
**
