# Supplementary material for: Example-based learning in heuristic domains: can using relevant content knowledge support the effective allocation of intrinsic, extraneous, and germane cognitive load?
Source: Front Psychol. 2024 Sep 23;15:1387095. doi: 10.3389/fpsyg.2024.1387095 (PMC11457169; doi:10.3389/fpsyg.2024.1387095)
Supplement: Supplementary file 6 [file Table_6.DOCX]

Supplementary Material F

Examples of the Course-Relevant Content Provided to The Two Groups

| Skill component | Integrated Into Video-based Worked Examples for the Relevant Content Group | Provided as Separate Text for the Irrelevant Content Group |
| --- | --- | --- |
| Instruction | On the next pages you will see three short videos that are intended to deepen the content of the learning text. In them, two friends, Anna (right) and Sofia (left), talk about [e.g.,] the consumption of violence-containing media and its effects on children's behavior. Please watch each video in its entirety before answering the questions below it […] | You will now be given a short learning text with information about [e.g.,] the consumption of violence-containing media and its effects on children's behavior. As you read this text, remind yourself of what you just learned about [e.g.,] epistemic beliefs. |
| Epistemic Beliefs | *Anna*: I think that the consumption of violence-containing media triggers aggression in children. After all, children see violence every day in a format that strongly resembles real life. After violence is normalized in the media, children learn that violence is normal and behave more aggressively. Therefore, violent movies and computer games should be banned.  *Sofia*: I understand what you mean, but I think that's too simplistic a view. After all, there are many ways in which violence-containing media and aggression could be related. For example, it would also be possible that children who are prone to aggressive behavior are more likely to consume violent movies and computer games. Or there could be a third variable that causes or influences the consumption of violence-containing media and aggressive behavior.  *Anna*: But what kind of factor is that supposed to be? And it doesn't make sense that only children who are already prone to aggressive behavior consume violent media. After all, violence in the media is now so widespread that all children are exposed to some degree of it. I still believe that the violence in the media also causes more aggressive behavior in real-life. […] | There has been much discussion in the media over the last two decades about whether the consumption of violence-containing media triggers aggression in children. After all, children see violence in a format that strongly resembles real life. After violence in the media is "normalized" to a certain extent, children may learn that violence is normal and behave more aggressively. […] Therefore, some people believe that violent movies and computer games should be banned. Violence is now so widespread in the media that all children are exposed to it.  However, it is not clear whether there is a direct causal link here. There are many ways in which violence-containing media and aggression could be linked. For example, it is also possible that children who are prone to aggressive behavior are more likely to consume violent films and computer games. Children who see a lot of violence in their environment or experience it themselves could also be more interested in violence-containing media and behave more aggressively. […] Or there is a third variable that causes or influences the consumption of violent media and aggressive behavior.  […] |
| MDL | […] Next, we take a closer look at what the arguments of each information source are and how they are substantiated. Here we fill in the blue boxes in our graph. In the first newspaper article from Der Spiegel, the main argument is that most parents and teachers find the Gymnasium important because they believe it is best for high-achieving students to be taught with other good students. This argument fits with my prior knowledge: From the perspective of high school students in the highest track, it is argued that the tracking in the school system should be maintained because otherwise these students get distracted or underchallenged. It is also stated in the article that students are most disturbed by distracting classmates and boring classes. This both suggests that tracking is good because higher-performing students can be better supported and challenged. The information is coherent within the article and fits the available data. | Germany uses a tracking school system, which means that children are divided into different types of school after elementary school. This system has been under discussion for a long time and there are proposals to abolish the tracking system. Therefore, it is important to examine the advantages and disadvantages of the tracking system.  Most parents and teachers believe that it is best for high-achieving students to be taught with other good students. Then students are less likely to be underchallenged and they are not distracted by their classmates. Students say they are most disturbed by distracting classmates and boring classes, according to one study. As a result, the tracking system is positive for high-achieving students. […] |
| Argumentative Thinking | […]  Anna: All right. I read a study once where it was shown that the same term paper received a very different grade from different teachers. So the grade was not an objective representation of the quality of the term paper. I would see that as evidence of my statement.  Sofia: And that bothers you?  Anna: Yes! It is always emphasized that the advantage of grades is that they are an objective feedback of student performance. But if I get a bad grade just because the teacher doesn't like me, then the use of grades is hurting me. Besides, as far as I know, the school law says that grades should be used to make students' performance more comparable.  Sofia: I see what you mean. So your principle is that grades harm children because they are not objective and children feel bad when they get a bad grade. They also don't serve their purpose of making student performance more objectively comparable.  Anna: Exactly!  Sofia: But is that always the case? Or are there only certain conditions under which grades are not objective?  Anna: Well, I've also read that some factors influence the assignment of grades. For example, children's social background and gender affect the grades their teachers give them.  Sofia: Okay, so grades are probably often not objective. Are there also exceptional conditions, where grades are assigned more objectively?  Anna: I could imagine that with anonymous grading, the grades would be fairer. […] | […]  Grades are supposed to provide objective feedback on student performance and thus make student performance more comparable. One view shared by some people, however, is that grades are not truly objective. For example, one study showed that the same assignment can be graded very differently by different teachers. Many students have the impression that the teacher's favorite students get better grades. […]  Several factors can influence the assignment of grades. For example, children's social background and gender affect the grades their teachers assign. Therefore, grades could harm children because children feel bad when they receive a bad grade.  On the other hand, measures are already being taken in some areas to make the awarding of grades more objective and thus fairer. For example, grades are sometimes awarded anonymously so that student characteristics do not influence the grading. |

**
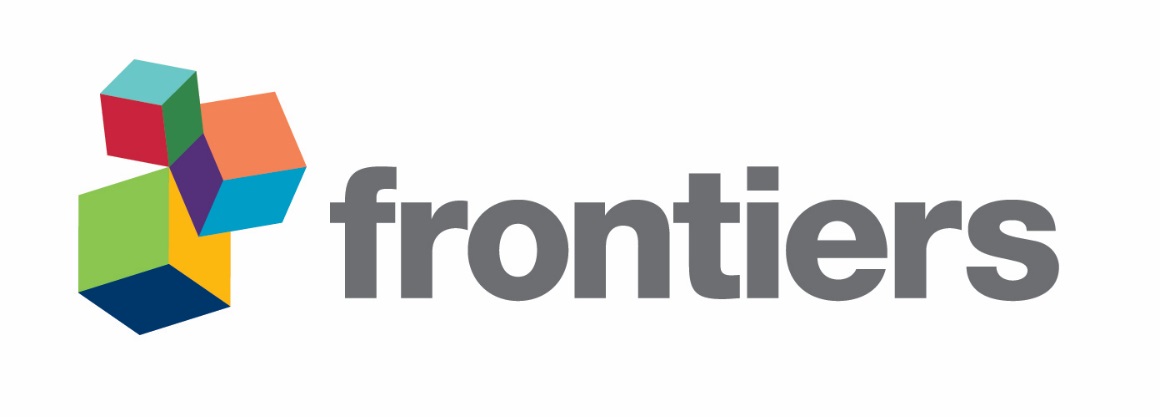
**
